# Role of Metabolic Acids in Shaping Bone-like Apatite Architectures

**DOI:** 10.1021/acs.chemmater.5c02593

**Published:** 2026-03-26

**Authors:** Yang Li, Rui Li, Thomas Kress, David G. Reid, Karin H. Müller, Danielle Laurencin, Christian Bonhomme, E. Alex Ossa, Chenglong Li, Robin van der Meijden, Nico Sommerdijk, Melinda J Duer

**Affiliations:** † Yusuf Hamied Department of Chemistry, 2152 University of Cambridge, Lensfield Road, Cambridge CB2 1EW, U.K.; ‡ Cambridge Advanced Imaging Centre, Department of Physiology, Development and Neuroscience, University of Cambridge, Downing Street, Cambridge CB2 3DY, U.K.; § ICGM, Univ Montpellier, CNRS, ENSCM, Montpellier 34293, France; ∥ Laboratoire de Chimie de La Matière Condensée de Paris, UMR Sorbonne Université CNRS, Sorbonne Université, 4, Place Jussieu, Paris 75252 Cedex 05, France; ⊥ Department of Production Engineering, 28008 Universidad EAFIT, Cra 49, No 7 sur 50, Medellín 050022, Colombia; # Department of Medical BioSciences and Radboud Technology Center—Electron Microscopy Center, 6034 Radboud University Medical Center, Geerte Grooteplein, Nijmegen 6525 AG, The Netherlands

## Abstract

Bone mineral forms both inside and between collagen fibrils in the extracellular matrix. While the morphology of intrafibrillar bone mineral has been hypothesized to be primarily controlled by the size and shape of the restricted spaces inside collagen fibrils within which the mineral forms, what controls the architecture of the extrafibrillar mineral is still an open question. While bone mineral is primarily apatitic in composition, it also contains significant quantities of cell respiration metabolites, in particular, carbonate, citrate, and lactate. An as-yet unanswered question is what, if any, role do these metabolites collectively play in determining the 3D architecture of bone mineral. Here, we propose a composite model of bone mineral that accounts for both intra- and extrafibrillar mineral environments, and to that end, we develop apatitic materials containing citrate and lactate or carbonate that mimic the densely packed ionic environments within which bone mineral forms in vivo. We find that incorporating citrate and lactate leads to complex mineral architectures reminiscent of those in extrafibrillar bone mineral, including mineral crystal curvature. Our results suggest that metabolic acids may play an important role in building the 3D architecture of extrafibrillar bone mineral.

## Graphical abstract

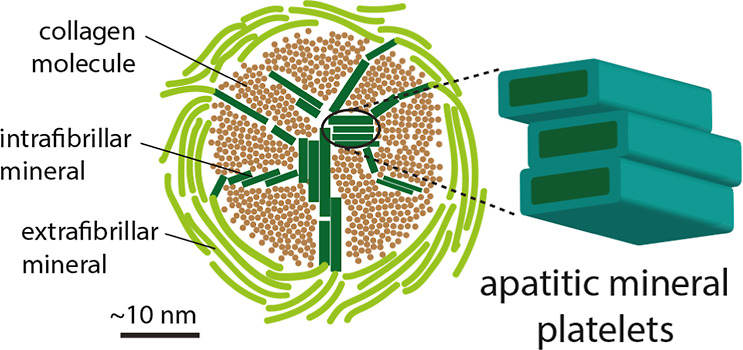

## Introduction

Bone mineral is arguably one of the most important chemical structures in the body. It forms in association with collagen fibrils in highly complex, dynamic chemical environments, containing many different ions and noncollagenous biomolecules. 3D-electron microscopy (3D EM) studies of bone show mineral both inside (intrafibrillar) and outside (extrafibrillar) collagen fibrils (Figure 1A). Extrafibrillar mineral in 3D EM appears as interleaved, nanoscopic, curved platelets that connect with intrafibrillar crystals, forming a cross fibrillar network.^1–3^ While the architecture of intrafibrillar mineral has been hypothesized to arise primarily from the templating effect of the collagen molecules that surround it,^3^ what controls the size and shape of the extrafibrillar mineral platelets is still an open question.

**Figure 1:**
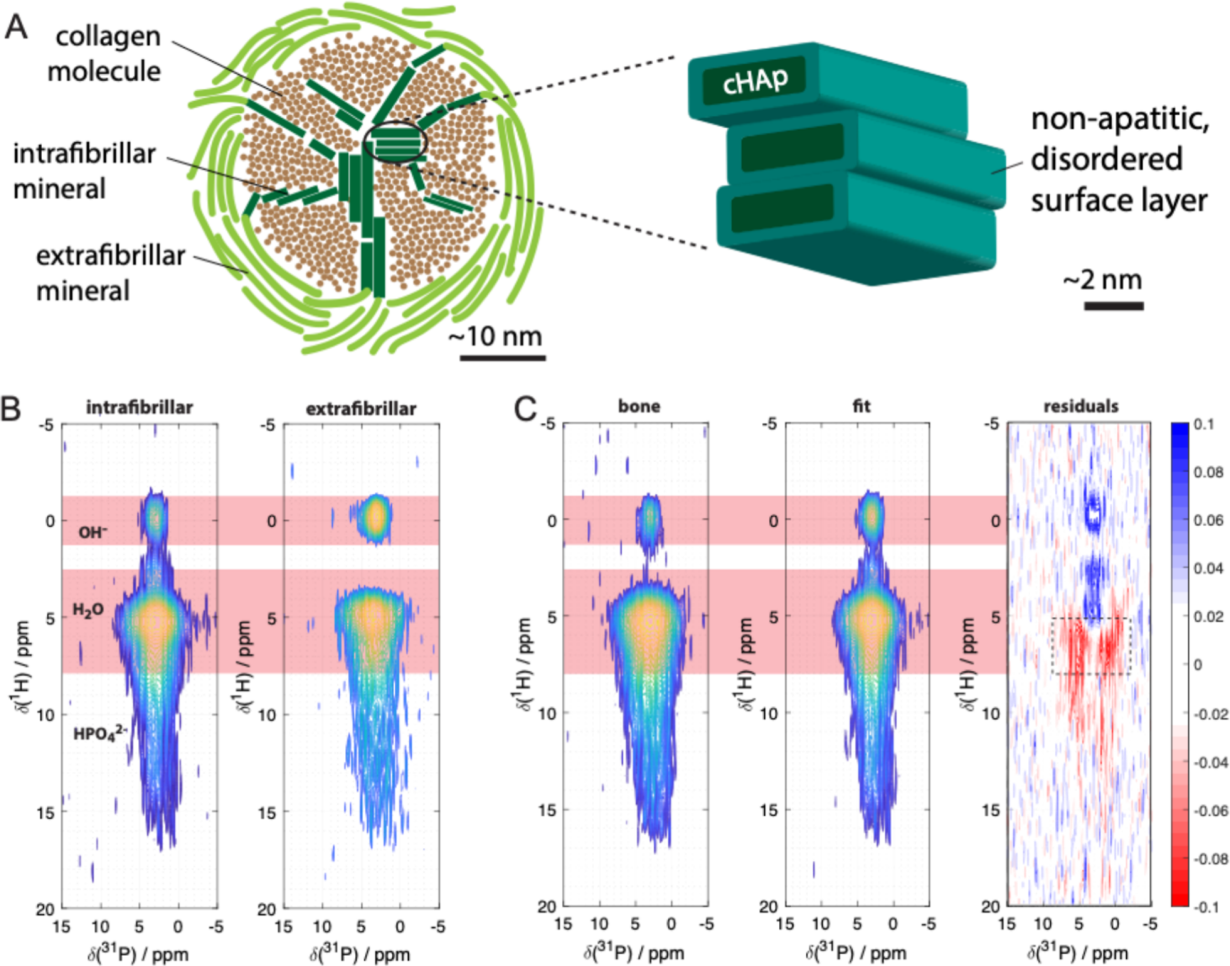
A). Schematic showing (left) the intra- (dark green) and extrafibrillar (light green) environments of bone mineral crystals with respect to collagen fibrils. A collagen fibril is represented in the cross-section, with collagen molecule cross-sections as brown circles. Right: schematic of the current model of the bone mineral platelet structure and composition. (B). 2D ^1^H–^31^P correlation NMR spectra (500 μs contact time) of in vitro samples of collagen fibrils calcified with (intrafibrillar) and without (extrafibrillar) cHAp. The expected spectral regions for mineral ^1^H signals are indicated. The region for mineral HPO_4_^2–^ ^1^H signals extends from ∼2 to ∼18 ppm, with HPO_4_^2–^ substituted into HAp expected ∼2∼7 ppm and surface HPO_4_^2–^ above ∼8 ppm.^24^ Relative total intensities of the two spectra are scaled so that the sum of the spectra gives the “fit” spectrum in C. (C). Best fit of the 2D ^1^H–^31^P correlation NMR spectrum (500 μs mixing time) of the air-dried, ground mouse limb bone by a linear combination of the spectra from intra- and extra-fibrillar mineral models in B. The difference between the bone and fit spectra (“residuals”, right) shows that there is significant signal intensity missing in the water/HPO_4_^2–^ region of the spectra of the mineral models, in particular in the water correlation signal indicated by the dotted rectangle.

The molecular structure of the mineral in bone has been extensively explored, in particular by solid-state NMR spectroscopy.^4–10^ The result of these studies collectively is a model for bone mineral of nanoscopic platelets comprising a core of crystalline hydroxyapatite (HAp, Ca_10_(PO_4_)_6_(OH)_2_) and surface layers of highly hydrated, disordered nonapatitic calcium phosphate, with ion substitutions in both core and surface regions, the most abundant being carbonate, a key cell respiration metabolite.^4,5^

The discovery of bone mineral platelet surfaces having a different composition to their core was an important step toward understanding the complexity of the bone mineral structure.^4,5^ The detailed composition and structure of the disordered surface layers is still the subject of intense debate and for good reason: the interleaving of bone mineral platelets found in 3D EM studies implies that neighboring mineral platelets interact via their surface layers.^1,5^ By extension, this suggests that these surface layers are likely to be major determinants of the bone mineral 3D architecture and, therefore, mechanical properties. The crystalline hydroxyapatitic core of bone mineral platelets is a universal motif throughout bone mineral, consistent with hydroxyapatite being by far the most thermodynamically stable calcium phosphate structure at physiological pH.^11^ However, the universality for the composition and structure of the nonstoichiometric mineral on platelet surfaces and interplatelet regions seems less likely. In a simple calcium–phosphate system, the calcium and phosphate ion concentrations and pH determine the thermodynamically most stable calcium phosphate phase, typically a composite or intimate mixture of crystalline stoichiometric and disordered nonstoichiometric components.^11^ This is the main underlying reason for the apatitic core—disordered surface anatomy of bone mineral platelets, and if the bone calcification environment contained only calcium and phosphate ions, the mineral platelet surface layer composition would depend only on the mineral ion concentrations, pH, temperature, and pressure, as for any phase diagram. However, the bone calcification environment contains other ions (many of which can be substituted into the HAp lattice) and many, possibly abundant, calcium-binding proteins and metabolites that distort the effective mineral ion concentrations in solution, thereby affecting the position of the calcification system on the phase diagram.

The calcium-binding proteins and metabolites also bind to forming mineral nuclei and crystals, and this, along with the physicochemical properties of the space available for the mineral, affects the kinetics of mineral formation. The kinetics matter because if the energy barrier to a mineral structure is high compared to *kT*, the probability of that structure forming is low, even if it is a thermodynamically stable structure compared with other possibilities. Thus, the mineral platelet surface structures in in vivo bone arise from a delicate balance of energetics and kinetics. Energetics drive toward surface layer structures that minimize the net free energy of the surface layer ions in their interactions with both the underlying hydroxyapatite core and their surroundings. Kinetics determine the rate at which any surface structure can form and thus are a significant factor in determining the extent to which different possible surface structures are represented in the final mineral composition. It is therefore reasonable to expect that the mineral platelet surface layer composition and structure can be variable and will depend on additional factors such as the ion, metabolite, and protein composition of the extracellular fluid and the physicochemical properties of the mineral environment.

Calcium-binding metabolites in the bone calcification environment not only perturb the effective calcium ion concentration but also become components of the bone mineral itself. Bone mineral contains significant quantities of cell respiration metabolites in addition to carbonate: citrate (2–5 wt %)^12–14^ and variable amounts of lactate^12,13,15^ are those that are currently known about. Their presence in bone mineral is consistent with the expectation that respiration metabolites are present in the extracellular fluid around the metabolically active cells (osteoblasts) that are driving mineral formation. The concentrations of citrate and lactate in bone vary significantly; for citrate, with age and disease, and for lactate, with blood plasma lactate concentration. For instance, the concentration of bone citrate is more than halved in osteoporotic and aged mice and rats compared to healthy controls,^16^ while lactate concentration in bone increases more than 6-fold in turtles between normoxic and anoxic conditions.^17^ In contrast, the calcium/phosphate ratio of bone mineral is almost invariant, including with age: 2.25 (mean value) in human neonates to 2.29 in adults, a less than 2% increase over 35 years of bone growth and turnover.^18^ These data imply that the hydroxyapatitic cores of bone mineral platelets change in composition relatively little with age, while the metabolite content can change considerably more. In turn, this suggests that the metabolic anions are incorporated primarily into nonapatitic disordered mineral regions rather than as substitutions into crystalline hydroxyapatite mineral platelets. This is consistent with citrate and lactate both being too large to be substituted into the hydroxyapatite lattice.^19^

The physicochemical environments for forming and maturing mineral can be broadly categorized into intra- and extrafibrillar mineral locations, and these two mineralizing regions provide significantly different physicochemical properties. Prior to mineralization, the extrafibrillar environment has high concentrations of proteoglycans containing charged, highly hydrophilic glycan chains plus associated water,^20^ and many calcium-binding proteins are likely present, in addition to the ionic and metabolite content of the extracellular fluid. In contrast, the intrafibrillar environment is characterized by highly restricted space, restricted numbers of water molecules, bounded and interspersed by hydrophobic, relatively rigid collagen protein, and likely only low concentrations of noncollagenous proteins. These highly different chemical environments offer the possibility of mineral platelets with highly different surface layers forming on them. However, bone mineral is currently typically modeled with a universal motif of nanocrystalline carbonate-substituted hydroxyapatite platelets, irrespective of the mineral’s intra- or extrafibrillar location.

In spite of their relative abundance in bone, the current model of bone mineral also does not include citrate and lactate, two components that are likely to be found primarily in the crucial interplatelet mineral and whose concentrations in mineral are two of the few parameters that correlate with age and bone pathologies associated with increased bone frailty. Here, we explore the extent to which nanocrystalline carbonated HAp can model intra- and extrafibrillar in vivo bone mineral and develop a bone mineral model in which intra- and extrafibrillar mineral are explicitly modeled and which incorporates the currently known metabolites that may contribute to bone mineral structure and health. The model we develop allows insight into how the properties of bone mineral may change with age and metabolic disease through altered concentrations of the metabolites that contribute to its structure and opens the potential for hypotheses about the molecular mechanisms behind dysfunctional bone mineral.

## Results

While the HAp core of bone mineral platelets seems likely to be a universal motif, the very different physicochemical environments in which intra- and extrafibrillar minerals form imply that the platelet surface compositions have the potential be significantly different in these two environments. Thus, we sought to explore model mineral systems in which we could separately model intra*-* and extrafibrillar mineral compositions and structures.

Given the widespread modeling of bone mineral as nanocrystalline carbonated HAp, we began by exploring the extent to which carbonated, nanocrystalline HAp models in vivo bone mineral. We generated samples of collagen fibrils containing primarily either intra*-* or extra*-*fibrillar carbonated HAp (cHAp) to model the two primary environments of bone mineral in vivo. Polyanions including polyAsp are known to promote intrafibrillar mineral formation, while the absence of polyAsp generates primarily extrafibrillar mineral. SEM was used to assess the spatial extent and homogeneity of mineralization across collagen fibrils, while TEM was used to examine mineral morphology at the nanoscale (Figure S1). SEM images of collagen fibrils for samples with and without the inclusion of polyAsp show the expected intra- and extrafibrillar mineral, respectively (Figure S1). As is typically found when using polyAsp in mineralization of collagen fibrils, images show some extrafibrillar mineral also forms but that polyAsp strongly biases mineral formation toward the intrafibrillar compartment. Images of the extrafibrillar mineral formed without polyAsp show disorganized and randomly located mineral crystals over the collagen fibrils, in contrast to the highly organized mineral crystals in in vivo bone, suggesting that collagen fibrils alone do not model the physicochemical environment in which extrafibrillar bone mineral forms in vivo.

We then compared the mineral molecular structures in these synthetic samples with those of ex vivo bone samples using 2D ^1^H–^31^P solid-state correlation NMR spectra. The projections of these spectra onto the ^1^H and ^31^P spectral axes are the spectra of the ^1^H and ^31^P environments of the mineral component in each case. The correlation signals in 2D correlation solid-state NMR spectra come from ^1^H and ^31^P that are physically close in space in the sample and which are in a “solid” state, i.e., in mineral and not, for instance, cell phospholipids. Only ^1^H that are spatially close (within ∼1 nm) to ^31^P are represented in the 2D ^1^H–^31^P correlation spectrum, including in the projections on the ^1^H axis. Signal intensities in these 2D spectra depend on how close in space the correlated ^1^H and ^31^P nuclei are and the length of the ^1^H to ^31^P cross-polarization time in the 2D pulse sequence. For a given cross-polarization time, the closer in space a ^1^H–^31^P pair is, the higher the intensity of their respective correlation signal is. To bias the spectral signal intensity toward signal intensity from interplatelet mineral regions, we used short ^1^H to ^31^P cross-polarization times in these experiments (500 μs). The interplatelet regions have been shown to be rich in hydrogen phosphate (HPO_4_^2–^, indicative of a disordered mineral structure) and highly hydrated, so that phosphate anions hydrogen bond to water molecules. These phosphate anions all contain short ^1^H–^31^P distances (2.2–3.6 Å) compared with the ∼5 Å ^1^H–^31^P distance for OH^–^—PO_4_^3–^ in hydroxyapatite. Thus, at short ^1^H–^31^P cross-polarization times, the 2D ^1^H–^31^P correlation signal intensities are dominated by signals expected from the disordered interplatelet regions rather than (crystalline) hydroxyapatite. Longer cross-polarization times (2 ms and greater) emphasize the crystalline hydroxyapatitic component, which is broadly similar throughout bone mineral and therefore not a sufficient discriminator for assessing the similarity of a mineral model to bone mineral.

To ensure that the mineral in the native bone sample was in a similar hydration state to that of the cHAp in vitro samples, all samples (bone and synthetic samples) were coarsely ground and air-dried for 24 h prior to the NMR experiments to remove the more loosely bound water. While it is possible to retain native hydration of intact bone samples during NMR experiments,^21^ the in vitro samples are powders that readily lose water when they are removed from the reaction vessel. Initial NMR experiments showed that the cHAp-calcified collagen lost water during the long NMR experiment times, even at low temperatures, compared with intact bone samples. The ratio of H_2_O: HPO_4_^2–^ NMR signal intensities is known to be significantly affected by the hydration state.^5^ While removing loosely bound water may result in the loss of some water strongly bound to mineral, with possible consequences for, e.g., the thickness of hydrated layers on cHAp crystal surfaces in synthetic and bone samples, ensuring all samples are in a similar hydration state is essential for the quantitative spectral comparisons that are our primary aim here. To ensure we have not removed significant amounts of mineral-bound water in any sample, we confirmed the NMR spectra for each sample retained^22^ ^,^ ^23^ the expected large water ^1^H signal from mineral-bound water, as discussed for each sample below.

Figure 1B shows the 2D ^1^H–^31^P correlation spectra for samples of intra- and extrafibrillar carbonated HAp (as synthesized, with intact collagen fibrils), which both show the expected correlation signals for (i) HAp OH–^1^H, (ii) disordered mineral water ^1^H, and (iii) HPO_4_^2^^–^^1^H. The spectra in the ^1^H dimension of these spectra are dominated by water ^1^H signals, showing that our drying process retains the expected the hydrated surface layers on cHAp particles.

We then performed a least-squares fit of a linear combination of the spectra for intra- and extrafibrillar cHAp in Figure 1B to the 2D^1^H–^31^P correlation spectra of mouse limb bone recorded under identical NMR and sample conditions; the spectrum in the ^1^H of the 2D spectrum for bone (Figure 1C) is also dominated by a water ^1^H signal, as observed previously,^23^ showing that bone mineral too retains the expected mineral-associated water after our air-drying process. The resulting spectral fit displayed in Figure 1C shows that significant spectral intensity is missing in the fitted spectrum in the spectral region associated with mineral-bound water and/or disordered mineral HPO_4_^2–^.

That only a subset of the water environments in bone mineral can be modeled by either form of collagen-cHAp implies that there is an additional water environment unrelated to the carbonated apatite platelets and the collagen–mineral interface water environments modeled by the collagen-cHAp materials. Thus, we hypothesized that there are some bone mineral platelet surfaces or interfaces with compositions that are not modeled by cHAp or association with collagen fibrils.

We have previously hypothesized that citrate anions may reside in the water-filled interfaces between bone mineral HAp platelets.^25^ The interplatelet gap between extrafibrillar mineral platelets in bone has been estimated to be < 1 nm.^1^ Citrate anions span 0.6–0.8 nm depending on their conformation and thus are of suitable size to be located in these mineral interplatelet gaps. By the same reasoning, lactate too can fit in the mineral interplatelet gaps, but neither anion can fit in the HAp lattice. Thus, we hypothesized that additional bone mineral water environments may come from interplatelet interfaces mediated by citrate and lactate. The interplatelet gaps are primarily a feature of the extrafibrillar mineral;^2,26^ intrafibrillar mineral has been reported as having a more uniform continuous morphology in bone.^2^ Moreover, while citrate and lactate are both small enough to penetrate inside collagen fibrils, it is unclear how high a concentration of either can be achieved inside fibrils and thus whether these anions could be present in high enough concentration in the intrafibrillar environment to significantly modify the bone intrafibrillar mineral structure. In contrast, citrate and lactate concentrations can be expected to be maximized in the extrafibrillar environment. Thus, extrafibrillar minerals can be expected to be a significant source of the citrate and lactate found in bone mineral. Our next step then was to design an extrafibrillar mineral model that contains nanoscopic platelets of HAp with interplatelet layers containing water, citrate, and lactate. To ensure that we model the physicochemical properties of the extrafibrillar environment and specifically to ensure we separate any chemical control of the mineral architecture by these metabolic anions from that of chemical functionalities on collagen fibril surfaces, the model we develop here is collagen-free.

To generate nanoscopic HAp platelets interleaved with hydrated layers containing citrate, we exploited two well-known features of calcium phosphate chemistry: the transformation of OCP to HAp through hydrolysis^27–33^ and the ability of OCP to incorporate carboxylic acid anions into its structure (Figure 2A).^19,25,34–44^ Carboxylic acid anions locate to the hydrated layer of OCP,^25,34,37,42,43,45^ where they bind to the apatitic-like structures on either side of the OCP hydrated layers.^25^ Transformation of HAp within an OCP lattice is hypothesized to begin in the apatitic layers of OCP.^32^ We reasoned that the presence of citrate and/or lactate in the OCP hydrated layers would inhibit transformation of some of these hydrated layers resulting in the retention of citrate, lactate, and water-containing layers between HAp layers or platelet-shaped structures.

**Figure 2:**
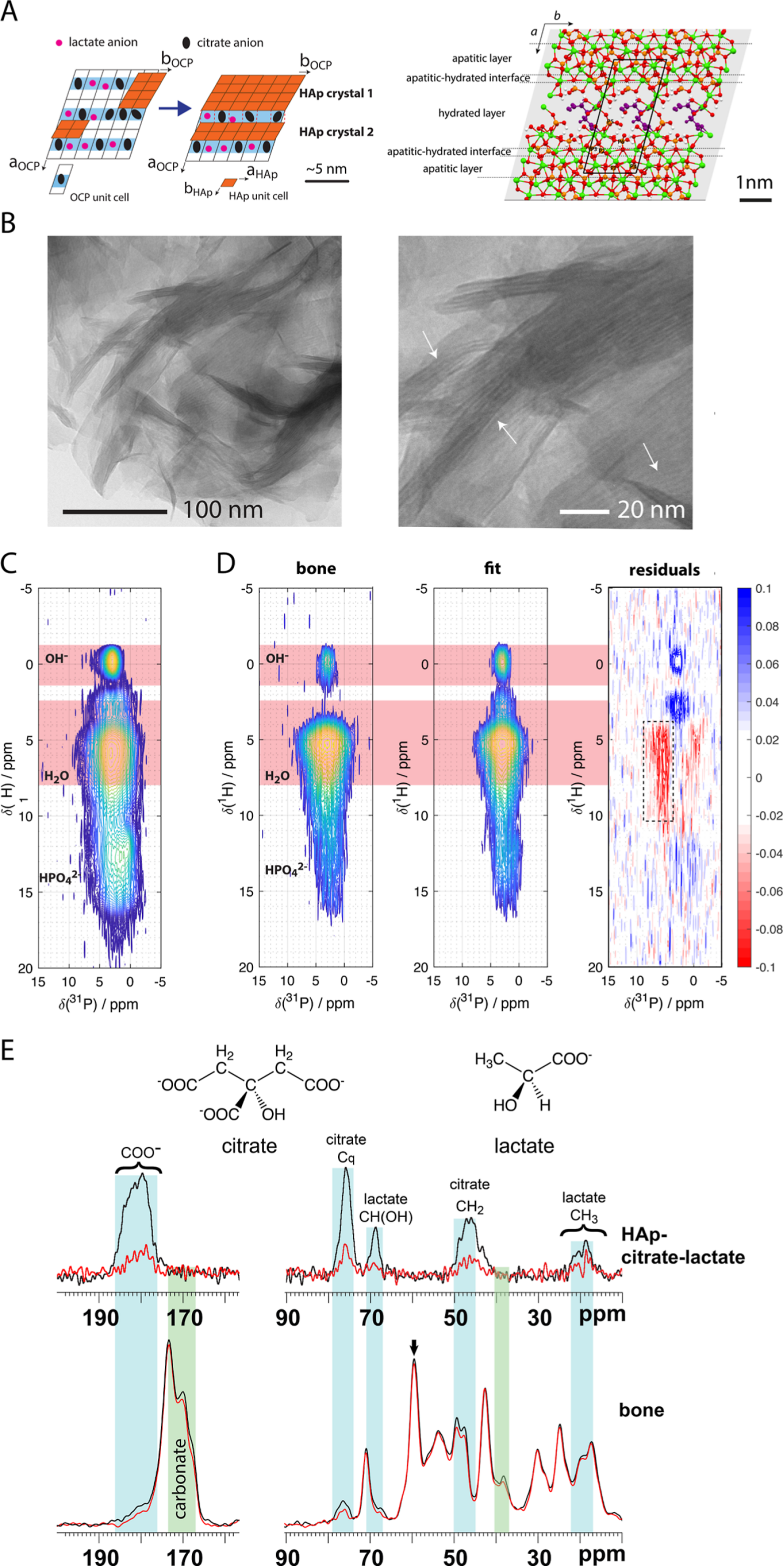
(A). Left: schematic of hypothesized transformation of an initial transient OCP–citrate–lactate phase to an HAp-containing material (orange). Full transformation of the OCP phase to HAp is inhibited by the presence of citrate (black circles) and lactate (smaller pink circles) in the lattice, both of which need to be expelled from the lattice in order for HAp to form. Some citrate and lactate thus remain in OCP-like hydrated layers (pale blue) between regions of the HAp structure. Right: the structure of the OCP–citrate double salt determined previously by NMR crystallography,^45^ showing the bridging of one citrate anion per unit cell across the hydrated layer of the OCP structure. (B). TEM images of the HAp–citrate–lactate mineral showing interlayering (white arrows). (C). 2D ^1^H–^31^P correlation spectrum of HAp–citrate–lactate (mixing time 500 μs). (D). Best fit of the 2D ^1^H–^31^P correlation NMR spectrum (500 μs mixing time) of air-dried, ground mouse limb bone by a linear combination of the spectra from intrafibrillar cHAp (see Figure 1C) and HAp–citrate–lactate. The difference between the bone and fit spectra (right) shows that there is signal intensity missing from a specific type of phosphatic site (dotted rectangle). (E). ^13^C­{^31^P} REDOR spectrum of HAp–citrate–lactate (dephasing time 100τ_R_, MAS rate 10 kHz) and horse bone (dephasing time 80τ_R_, MAS rate 12.5 kHz). The dephasing time for bone was chosen to minimize dephasing on collagen signals to highlight the REDOR dephasing on noncollagen signals. Colored bars indicate the main spectral regions with REDOR dephasing; blue bars are regions where there is REDOR dephasing in the spectra for both bone and HAp–citrate–lactate; and green bars are regions where there is no corresponding dephasing in the HAp–citrate–lactate spectrum. Note that the bone spectrum here is dominated by signals from collagen and that any signals from metabolites in the bone mineral are expected to be small in comparison.

Thus, we prepared citrate- and/or lactate-containing OCP-carboxylic acid double salts^42,45^ and converted these into the equivalent apatitic minerals via hydrolysis (Table S1). The synthesis for each material was repeated at least four times and found to give reproducible compositions, TEM images, and NMR spectra, as described below. We focus here on the material from the synthesis with both citrate and lactate, HAp–citrate–lactate; further analysis of the citrate-only and lactate-only materials can be found in the Supporting Information. TEM images of the HAp–citrate–lactate material showed curved, leaflet-like particles with interleaved fingers of electron-dense regions (1.2–2.4 nm thick) and less electron-dense regions (0.9–1.3 nm thick) (Figure S1) consistent with the expected interlayering from the OCP–carboxylic acid to HAp–carboxylic acid transformation, while single area electron diffraction (SAED) on the same samples showed patterns consistent with polycrystalline hydroxyapatite (Figure S1). Solid-state ^31^P­{^1^H} cross-polarization magic-angle spinning (CPMAS) NMR spectra of HAp–citrate–lactate (Figure S2) confirmed formation of nanocrystalline HAp with a dominant, broad signal centered at ∼3 ppm, characteristic of nanocrystalline HAp, and exhibited loss of characteristic OCP signals, as expected for the OCP–carboxylic acid to HAp–carboxylic acid transformation (see Figure S2 and the associated text). Powder X-ray diffraction (pXRD) also showed the expected loss of the OCP-like (100), (110), and (010) reflections, and the pXRD pattern for HAp–citrate–lactate overall resembled that of bone mineral (Figure S3). Thus, we concluded that the synthesis strategy resulted in substantial transformation to HAp nanocrystals interleaved with hydrated layers as we had designed for and, moreover, that the resulting HAp–citrate–lactate material showed crystal curvature mimicking that additional feature of bone mineral as well.

We first explored the extent to which the molecular structure of HAp–citrate–lactate models that of bone mineral using ^43^Ca NMR spectroscopy (Figure S5). ^43^Ca chemical shifts are fairly sensitive to Ca local environments and, hence, are excellent reporters for the similarity between the mineral structures present in our materials and bone mineral.^44,46–49^ Interestingly, the ^43^Ca spectrum for HAp–citrate–lactate maps onto a considerable proportion of the ^43^Ca spectrum for bone, in contrast to the ^43^Ca NMR spectrum of HAp^49,50^ and carbonate-substituted HAp, which does not replicate the ^43^Ca NMR spectrum for bone mineral well.^56^ Specifically, the ^43^Ca spectrum for HAp–citrate–lactate here (recorded at 20 T) shows a signal maximum at −2 ppm and a line width at half height of 1.5 kHz, relatively similar to the −0.5 ppm peak maximum and 1.5 kHz line width for equine bone recorded at 18.8 T;^46^ the lower field in this case is expected to result in a less negative shift and lower line width compared to our data recorded at 20 T. This suggests that the distribution of Ca chemical environments in HAp–citrate–lactate mimics a significant proportion of those in bone mineral, consistent with HAp–citrate–lactate modeling extrafibrillar bone mineral.

We then recorded 2D ^1^H–^31^P correlation spectra of the HAp–citrate–lactate material (Figure 2C; see Figure S6 for 2D ^1^H–^31^P of the HAp–lactate and HAp–citrate materials) using the same short ^1^H–^31^P cross-polarization time as for the cHAp-collagen and bone samples previously. As for the cHAp samples, the 2D spectra are dominated in the ^1^H dimension by signals from water ^1^H, confirming that air-drying the samples to allow quantitative comparison between spectra retains the expected mineral interlayer water. We subsequently performed a least-squares fit of a linear combination of the 2D ^1^H–^31^P correlation NMR spectra for the HAp–citrate–lactate material to represent extrafibrillar mineral and intrafibrillar cHAp (Figure 1B) to the same mouse bone mineral 2D ^1^H–^31^P correlation spectrum as in Figure 1C. The resulting fit, shown in Figure 2D, is an improvement on the fit of intra- plus extrafibrillar cHAp (total residual 4.6 compared to 5.1 for the cHAp-only fit; see the Experimental Section for definition). In particular, inclusion of the HAp–citrate–lactate spectrum accounts for a substantial part of the “missing” mineral water signal from the cHAp-only fit in Figure 1C. The remaining ^1^H–^31^P signal intensity under-represented by this model comes from a ^31^P component centered around 5.5 ppm, as indicated by the dotted lines in the middle spectrum of Figure 2D. A least-squares fit to a linear combination of intrafibrillar cHAp, HAp–citrate–lactate, and extrafibrillar cHAp did not improve the fit and could also not account for the under-represented ^1^H–^31^P intensity centered at (∼6, ∼5.5 ppm) (Figure S7). Previous work has assigned ^31^P NMR signals around 5.5 ppm in hydroxyapatite to surface HPO_4_^2–^ anions,^24^ and a ^1^H chemical shift of 6 ppm corresponds to strongly bound (mineral surface) water. There are several reasons why such a site might be under-represented in our chemical model of bone mineral. Our extrafibrillar HAp–citrate–lactate mineral models the stacking of HAp nanoscopic platelets in bone mineral and the interplatelet region of mineral between the largest HAp platelet faces but not the interactions between the smaller HAp platelet faces, which in in vivo bone are likely exposed to the proteins and glycans that reside between collagen fibrils in bone and to other mineral platelets. Moreover, the HAp layers in our HAp–citrate–lactate model are 1.2–2.4 nm thick, while HAp platelets in bone are typically 4–6 nm thick^1,2^ and so can present significantly larger side faces, which will undoubtedly have HPO4^2–^ and water on their surfaces. We would expect the thinner HAp platelets in our HAp–citrate–lactate model to under-represent these chemical environments on bone HAp platelet side faces.

We can conclude then that a combination of our intrafibrillar cHAp and HAp–citrate–lactate broadly models the phosphatic environments of ex vivo bone mineral and that HAp–citrate–lactate models extrafibrillar bone mineral in terms of its curved, stacked platelet morphology and calcium, phosphate, and water chemical environments.

We next asked the question, do the citrate and lactate anions in the HAp–citrate–lactate material model those in bone mineral? ^13^C CPMAS NMR spectra of HAp–citrate–lactate (Figure 2E) show clear signals from citrate and lactate anions, demonstrating that these anions remain in the material as expected (see also Figure S8). We then compared the mineral environment of citrate and lactate in HAp–citrate–lactate with that for these anions in bone using ^13^C CPMAS and ^13^C­{^31^P} REDOR NMR spectra. ^13^C­{^31^P} REDOR spectra of bone allow us to identify the ^13^C signals from the carbon-containing molecules in close proximity to ^31^P, i.e., contained in bone mineral. In the REDOR spectra, the spatial proximity of ^13^C nuclei to phosphate ^31^P is identified by reduction of the ^13^C signal intensity between the reference and REDOR spectra for the sample (Figure 2E). The ^13^C signals from bone that exhibit a REDOR effect are highlighted in Figure 2E with colored bands. The REDOR signals at 47–49 and 75 ppm have been previously assigned to mineral-associated citrate. ^13^C signals from mineral-associated lactate have yet to be identified. The five ^13^C signals from HAp–citrate–lactate have chemical shift ranges that are highly similar to five out of seven of the bone REDOR signals; two ^13^C signals from citrate CH_2_ and C–OH ^13^C (46–50, 75 ppm, see Figure S8), two (17 and 68 ppm, see Figure S7) from the lactate methyl and C–OH ^13^C in HAp–citrate–lactate, and the overlapped HAp–citrate–lactate lactate and citrate carboxylate ^13^C signals (178–184 ppm). The similarity of the ^13^C chemical shifts for citrate and lactate in HAp–citrate–lactate and bone mineral means that we can conclude that the citrate and lactate environments in HAp–citrate–lactate are highly similar to those in bone. Of the remaining two ^13^C signals affected by REDOR dephasing, that in the region 167–173 ppm is from carbonate in bone mineral, a known component of bone mineral as described above, and the other at 38 ppm is yet to be definitively assigned, but we have previously shown that OCP–citrate^45^ has a ^13^CH_2_ signal in this spectral region (see also Figure S8), and it is possible that alternative citrate environments in bone mineral may account for this REDOR dephasing.

Finally, we asked, what are the citrate and lactate locations in the HAp–citrate–lactate material? Given the evidenced similarity of the citrate and lactate environments between HAp–citrate–lactate and bone mineral found by NMR, elucidating the citrate/lactate environment in the in vitro material will allow us to hypothesize the citrate–lactate location in bone mineral. We hypothesized that the citrate and lactate anions are in the hydrated interlayers between the HAp layers in HAp–citrate–lactate. To test this hypothesis, we compared the magnitude of ^13^C­{^31^P} REDOR dephasing for the HAp–citrate–lactate material and OCP–citrate and OCP–lactate double salts, in which we know the metabolic anion is in the OCP hydrated layer, between apatitic layers, i.e., in an environment that closely mimics the hypothesized environment of citrate and lactate in HAp–citrate–lactate. If the mineral environments of citrate and lactate in HAp–citrate–lactate and the OCP double salts are similar, we expect a similar magnitude of the REDOR effect on the citrate/lactate ^13^C signals. The alternative environment for citrate and lactate in HAp–citrate–lactate is on the outer surface of an HAp-hydrated layer stack, bound to the outermost HAp layer or to the small HAp side faces at the outer edges of HAp layers. These alternative environments would put citrate and lactate anions adjacent to a single HAp layer rather than between two HAp layers and would result in a REDOR dephasing effect of approximately half the magnitude. All ^13^C signals for the HAp–citrate–lactate material exhibit a substantial REDOR dephasing (Figure 2E), at least as large as that for the OCP–citrate and –lactate double salts for the same REDOR dephasing time (Figure S8), and so, we can conclude that these anions in the HAp–citrate–lactate material reside in the hydrated interlayers between the HAp layers and not on the outer surfaces of HAp-hydrated layer stacks. The environment in bone mineral that most closely resembles the hydrated interlayers between HAp layers is the interface regions between mineral platelets, which previous NMR studies show are highly hydrated also.^5^

## Discussion

Electron tomography studies^1–3^ on adult human bone show that there is mineral both inside (intrafibrillar) and between (extrafibrillar) collagen fibrils. There is compelling evidence from these studies that the intrafibrillar mineral morphology is molded by the confined space between fibril’s collagen molecules.^3^ The extrafibrillar environment is much less spatially restricted, so it is perhaps surprising that extrafibrillar mineral exhibits a highly complex architecture: nanoscopic, interleaved curved bone mineral platelets encircling collagen fibrils and extending across several fibrils typically. Clearly, there must be complex chemistry operating in the extrafibrillar space that can achieve this detailed 3D architecture. Equally, this chemistry likely differs from that in the intrafibrillar environment, if only because the extrafibrillar environment is characterized by the relative lack of collagen molecules, while the intrafibrillar environment is collagen-rich. Thus, in this work, we have developed a basis to model both the intrafibrillar and extrafibrillar chemical environments.

The extrafibrillar chemical environment in which bone mineral forms necessarily includes cell respiration metabolites such as carbonate, citrate, and lactate in the extracellular fluid. Whether the full range of extracellular metabolites also exists at the same concentrations inside collagen fibrils is yet to be determined, but they certainly pervade the extrafibrillar space and will therefore contribute to the complex phase diagram for mineral formation in the extrafibrillar space. Carbonate, citrate, and lactate are known to be present in bone mineral^12–15^ and are respiration metabolites that can be expected in the extracellular fluid around the metabolically active cells driving bone mineralization. The key questions then are where do these metabolites reside in the mineral structure, and what do they contribute to that structure? Carbonate is known to substitute for both orthophosphate and hydroxyl anions in HAp, including the apatitic regions of bone mineral.^5,8,51–53^ In synthetic HAp materials at least, carbonate plays a role in controlling the size of HAp domains through the lattice defects its incorporation causes, typically resulting in nanoscopic HAp crystals.^5,54,55^ Carbonate is a small anion, highly likely to freely diffuse into collagen fibrils wherever water and mineral ions can diffuse. Thus, our model of intrafibrillar mineral here is collagen fibrils mineralized with cHAp (using pAsp to ensure intrafibrillar mineral formation). The resulting material has mostly intrafibrillar and some extrafibrillar nanoscopic mineral platelets broadly aligned with the collagen fibril long axis, mimicking the organization of intrafibrillar minerals in bone. Our NMR analysis of this intrafibrillar mineral model shows that the nanoscopic mineral platelets have a significant contribution from highly hydrated, disordered, nonapatitic components, as is consistently found in studies of nanoscopic, carbonated hydroxyapatite without collagen,^5,56^ and which are consistent with the disordered mineral platelet surfaces hypothesized to exist throughout bone mineral. In contrast to carbonate, citrate, and lactate anions are too large to substitute for HAp anions. Whether they are also too large to freely diffuse into collagen fibrils with mineral ions is yet to be determined, and future research directions should investigate the extent to which citrate and lactate can impact the intrafibrillar mineral structure and composition.

In contrast, citrate and lactate concentrations in the extracellular fluid of the extrafibrillar environment are largely controlled by cell metabolism and can therefore be significant when extrafibrillar mineral forms, in particular citrate, as mineralizing osteoblasts are professional citrate-producing cells. It has been previously proposed that citrate may bind to mineral platelet surfaces^57^ or between mineral platelets,^45^ stabilizing the interplatelet hydrated layer. The possible roles for lactate in bone mineral have been less-studied, but comprehensive studies in freshwater turtles have demonstrated that bone mineral has significant capacity for lactate under anoxic conditions.^17^ Thus, we have developed here an extrafibrillar mineral model, HAp–citrate–lactate, containing citrate and lactate in hydrated layers between nanoscopic HAp platelets. The platelets in the HAp–citrate–lactate and HAp–citrate materials were typically curved (Figures 2 and S4) mimicking a characteristic aspect of both the intrafibrillar and extrafibrillar parts in the cross-fibrillar network of bone mineral crystals.^1,3^ Neither HAp-lactate (Figure S4) nor carbonate-containing nanocrystalline HAp display this essential crystal curvature,^5^ suggesting that the platelet curvature may be a result of citrate incorporation. Possible reasons for this include the citrate anion having different possible conformations. We have previously shown in OCP–citrate that different citrate conformations support different thicknesses of hydrated OCP layers.^25^ The same is likely to be the case of the interlayered HAp–citrate–lactate material, with the potential for the hydrated interlayers having variable thickness across individual layers, which could result in curvature of the layers and HAp layers on either side. Another possible reason for citrate inducing mineral crystal curvature is its ability to act as a pH buffer. The transformation from OCP-like phases to HAp requires loss of H^+^ from the initial OCP-like lattice. The presence of citrate anions with a significant capacity to bind H^+^ may have local effects on the rate of phase transformation, resulting in heterogeneous rates of HAp formation, which may affect HAp layer morphology. Whether such processes are relevant in bone mineral is a further question to be answered by future research, but our work here shows that citrate may have the capacity to facilitate bone mineral crystal curvature.

It has previously been proposed that monolayers of calcium citrate tetrahydrate can form preferentially between some HAp crystal faces.^58^ The authors of that study^58^ proposed an intriguing model in which such monolayers forming between preferential HAp crystal faces causes specific aggregation patterns of HAp crystals and suggested that such a model may account for the 3D architecture of bone mineral. The distance between stacked HAp platelets within a bone mineral platelet has been estimated to be less than 1 nm,^1^ which strongly suggests that the molecules that maintain the space between the platelets must be relatively small and not HAp-binding proteins, for instance. The citrate-/lactate-containing hydrated interlayers in our HAp–citrate–lactate material fulfill the <1 nm thickness condition. The citrate anion being 0.6–0.8 nm in span (depending on its conformation) would seem to be the primary feature for maintaining distance between HAp platelets in the HAp–citrate–lactate material and, therefore we hypothesize, also in bone mineral. If citrate is important in governing the bone extrafibrillar mineral 3D architecture, we would expect that citrate is also important for bone mechanical properties, given the relationship between 3D structure and mechanical properties. Early evidence for this comes from osteoporotic and aged mice and rats, which were found to have significantly lower bone citrate content than in controls.^59,60^

We have not explored here incorporating carbonate as a component in the extrafibrillar mineral or citrate and lactate in the intrafibrillar mineral model because our aim was to investigate the possible roles of citrate and lactate on the bone mineral structure as anions known to be present in bone mineral but whose roles are yet to be elucidated. It will be interesting in future work to understand the range of concentrations of citrate and lactate inside collagen fibrils and their possible effects on the intrafibrillar mineral structure and morphology. Similarly, it will be interesting to understand how carbonate anions compete with citrate and lactate in their incorporation into extrafibrillar bone mineral as carbonate anions have been shown to be present in the disordered, hydrated surfaces of nanocrystalline carbonated HAp, as well as substituting for anions in the HAp lattice. There are many other mineral ion substitutions in bone mineral HAp to consider as well as carbonate, in particular, cationic substitutions such as Na^+^ and Mg^2+^.^51^ These may have effects on the disordered, hydrated surface layer stabilities and, indeed, may be incorporated into these layers. We note that our SSNMR analysis showed that our current model of intrafibrillar cHAp and extrafibrillar citrate-/lactate-containing interlayered HAp is missing an HAp surface site (HPO_4_^2–^ bound to water), highlighting the true complexity of bone mineral—and likely the importance of other nonapatitic mineral ions in the bone mineral structure.

We also prepared HAp–citrate and HAp–lactate samples using the same synthesis protocol as that for HAp–citrate–lactate. The HAp–lactate material is a soft, chalky like substance, very different from the materials containing citrate, which are much harder, properties that we confirmed with AFM (Figure S9). These different mechanical textures and stiffnesses imply that the nature of the metabolic anion can have a significant impact on the strength of binding between HAp layers, either directly through the strength of bonding between the metabolic anion and ions in the HAp platelet surface or through affecting the extent of hydration of the interplatelet gap, or both. Intriguingly, fetal bone contains very high quantities of lactate compared to citrate, perhaps as a result of the more hypoxic environment within fetal tissues than in mature tissues.^61^ Foetal bones are necessarily soft to allow for the birth process, and we propose that the high lactate content of fetal bones is necessary to keep the bones soft until postpartum and that lactate may achieve this by inhibiting the transformation toward HAp, resulting in smaller HAp domains and larger disordered mineral regions (see Figures S4 and S6 and associated notes).

Bone mineral is regularly remodeled in vivo, a process involving the removal of bone mineral through acidic conditions created locally by osteoclasts. To gain some initial understanding of how the incorporation of citrate or lactate into bone mineral may affect bone turnover, we assessed the solubility of HAp–citrate, HAp–lactate, and ex vivo bone at pH 4 (Figure S10). As shown in Figure S10, under the conditions of the study (pressed powders of each material in aqueous conditions), HAp–citrate and bone mineral have similar solubilities, while HAp–lactate is significantly more insoluble than either. This suggests that the profile of metabolic acids in bone mineral could have significant consequences for bone turnover.

Increasing prevalence of bone pathologies associated with aging and metabolic diseases have highlighted connections between bone cell metabolism and the molecular structure of bone mineral. A model in which the 3D architecture of (at least) extrafibrillar bone mineral is governed by metabolic-acid-rich material between specific surfaces of HAp platelets can potentially explain how the bone mineral 3D architecture and mechanical properties are influenced by bone cell metabolism and potentially whole animal metabolism. The production of both citrate and lactate in bone is in part determined by the glucose and oxygen supply^12,13,62^ and partly by a delicate balance of hormones and growth factors known to be important in bone health,^12,63,64^ including insulin, estradiol,^12^ and parathyroid hormone. The phase diagram for calcium phosphate–carbonate–citrate–lactate–water is likely highly complex, and it is possible that even subtle changes in relative and absolute concentrations of carbonate, citrate, and lactate in vivo result in significantly different size distribution of HAp regions and composition and spatial arrangement of nonapatitic mineral regions, with significant consequent implications for bone mineral mechanical properties. Reduction of citrate/lactate concentrations during bone mineral formation would be expected to lead to larger HAp domains, based on our findings here. Bone HAp crystals are known to increase in size and crystallinity (atomic order) with animal aging, regardless of the amount of mineral or collagen per unit volume of the bone tissue, and the increasing crystal size and crystallinity correlates with increasing bone fragility.^65^ This important phenomenon is yet to be explained. We hypothesize that this bone aging characteristic results from complex changes in carbonate/citrate/lactate concentrations when bone is remodeled due to inevitable changes in osteoblast and/or whole-body metabolism with aging.

There are many other calcium-binding metabolites that could also be present during bone mineral formation and that may also affect the bone mineral structure. A further interesting possibility is the incorporation of pathological metabolites into bone mineral, pathological in the sense that their incorporation drives aberrant mineral architectures. We have previously shown that succinate, another product of the citric acid cycle, under the same reaction conditions as those used in this work, forms an OCP–succinate double salt, which is highly crystalline, forming large crystals compared to OCP–lactate and OCP–citrate, with ordered succinate anions bridging the OCP hydrated layer.^42^ While elevated temperatures induce transformation of OCP–succinate to HAp,^66^ incubation in our OCP hydrolysis reaction conditions at 37 °C for 10 days did not. This suggests the intriguing possibility that some metabolic anions may be pathological to bone mineral formation in that they inhibit the necessary phase transformations and stabilize pathological mineral structures. In turn, this suggests that the dysregulation of osteoblast and whole-body metabolism prevalent in aging could be a significant factor in pathologies associated with bone aging.

## Conclusions

From our work here, we propose that the presence of multiple metabolic acid ions during mineral formation may make significant contributions to the intricate 3D architecture of bone mineral. The presence of the metabolic acid anions, citrate and lactate, results in the formation of complex nanocrystalline apatitic phases where HAp nanocrystals interface through disordered, hydrated metabolic anion-containing interfaces. The presence of citrate generated curved mineral crystals in our model system, and the potential of citrate anions to promote crystal curvature may be relevant in the shaping of bone mineral to comply with their position in and around the collagen matrix in in vivo bone. We hypothesize that other cell metabolites may also be involved in forming the 3D architecture of bone mineral in vivo and thus the mechanical properties of bone mineral. A model in which cell metabolites contribute to control the 3D bone mineral architecture and calcium phosphate composition can potentially explain why bone mineral properties change in aging and pathologies such as osteoporosis. However, our model does not take account of the plethora of proteoglycans and noncollagenous proteins that are likely to be present in the bone mineralization environment, at least some of which are likely to also influence the bone mineral architecture. These proteins along with low abundance mineral ions not considered in this work, such as Na^+^ and Mg^2+^, also add further complexity to the bone mineral phase diagram and thus the detailed mineral composition that forms at any point in a bone’s lifetime. Further investigations on the effects of other cell metabolites on the bone mineral structure and their interplay with other mineral ions and the noncollagenous proteins in the bone extracellular matrix are urgently needed to understand the key players driving the important changes in the bone mineral structure with aging and disease.

## Experimental Section

### Synthesis

#### Intrafibrillar Mineral

Bovine type-I collagen scaffolds were cut into ∼5 × 5 × 5 mm cubes and soaked in pH 8 Milli-Q water for 1 h. The cubes were placed in tubes containing a mineralization solution and incubated at 37 °C for 7 days. The mineralization solution was prepared by mixing equal volumes of 6.8 mM CaCl_2_, 4 mM K_2_HPO_4_, 400 μg/mL poly­(Asp), and 3.4× PBS (composed of 465.43 mM NaCl, 9.12 mM KCl, 6.49 mM KH_2_PO_4_, and 29.33 mM Na_2_HPO_4_·H_2_O). The 3.4 *x* PBS solution was first supplemented with 100 mM KHCO_3_ before mixing. The final solution concentration was 1.7 mM CaCl_2_, 1.0 mM KH_2_PO_4_, 100 μg/mL polyAsp, and 25 mM KHCO_3_ in 0.8× PBS. All materials were dissolved in Milli-Q water, and the pH of the PBS was adjusted to 7.1 before mixing. The mineralization solution was replaced daily. After 7 days, the collagen scaffolds were centrifuged, washed with pH 8 Milli-Q water for 3 min, and vacuum-dried for 24 h.

#### Extrafibrillar Mineral

The conditions for extrafibrillar mineralization were identical to those used for intrafibrillar mineralization except that poly­(Asp) was not added to the mineralization solution.

The samples of intrafibrillar and extrafibrillar minerals were first sliced into 100 μm thick sections using a Leica VT1000S vibratome with a steel blade. Slicing was performed with the speed and vibration frequency set to 3.5 and 3, respectively. The middle slice was used for SEM and TEM imaging.

#### OCP–Citrate Double Salt

A solution of citric acid (2.643 g, 13.8 mmol) in HPLC-grade water (55 mL) was maintained at 37 °C, and the pH was adjusted to 5.52 by dropwise addition of conc. NaOH solution. α-TCP (836.1 mg, 2.70 mmol) was then added, and the pH of the resulting suspension was adjusted to 6.50 by dropwise addition of conc. NaOH. The suspension was stirred at 200 rpm for 48 h, after which the solid was isolated by gravity filtration and dried in air overnight at room temperature. The final product was a white powder (215 mg).

#### OCP–Lactate Double Salt

Sodium-l-lactate (2.8 g, 25 mmol) was dissolved in 130 mL of distilled water maintained at 37 °C, and then, α-TCP (1.6 g, Sigma-Aldrich) was added while stirring. The pH was adjusted to 6.50 by the dropwise addition of concentrated HCl solution. The reaction mixture was covered and stirred at 37 °C and 500 rpm for 24 h, after which the product was isolated by gravity filtration, washed with distilled water, and dried in air overnight at room temperature. The final product was a white powder (1.01 g).

HAp–lactate and HAp–citrate materials were synthesized as above except that the reaction mixture in each case was stirred at 300 rpm for 10 days (at 37 °C).

#### HAp–Citrate–Lactate

A solution of citric acid (1.32 g, 6.9 mmol) and sodium lactate (1.4 g, 12.5 mmol) in distilled water (100 mL) was maintained at 37 °C, and the pH was adjusted to 5.50 by dropwise addition of NaOH. α-TCP (1.60 g, 5.3 mmol) was then added, and the pH was adjusted to 6.50 by dropwise addition of NaOH. The suspension was stirred at 300 rpm for 10 days, and the solid was collected by gravity filtration and dried in air. The final product was a white powder (0.62 g).

All samples were air-dried for 24 h prior to characterization measurements.

C,H elemental analysis was performed with an Exeter Analytical CE440 elemental analyzer with combustion at 975 °C. Ca and P elemental analyses were measured with a Thermo Scientific 7400 ICP-OES instrument at 396.84 and 178.28 nm, respectively.

### Bone Samples

Femora were excised from 3 week C57BL/6 wild-type male mice and euthanized for purposes beyond this project. Bone marrow was first removed by excising the proximal and distal metaphyses, and the diaphysis was centrifuged briefly at room temperature. The remaining sample was snap frozen in liquid nitrogen and stored at −80 °C before NMR analysis.

### Solid-State NMR (SSNMR) Spectroscopy

A Bruker 400 MHz Avance spectroscopy II spectrometer was used for solid-state ^1^H, ^13^C, and ^31^P NMR measurements, at frequencies of 400.42, 100.6, and 162.1 MHz respectively, with standard Bruker wide-bore double and triple resonance, MAS probes unless otherwise stated. Samples were packed into disposable HR-MAS inserts where necessary (small sample volumes) and loaded into the 4 mm zirconia rotors for magic angle spinning (MAS), at a rate of 10 kHz, unless otherwise stated.

Samples were characterized using ^31^P direct polarization (DP), typically using a 2.5 μs ^31^P (90°) and 600 s recycle delay. ^31^P {^1^H} CP spectra for OCP–citrate–lactate were recorded with ^1^H 90° pulse length, 2.5 μs, ^31^P 90° pulse length, 2.57 μs, ^1^H–^31^P CP contact time, 1 ms, and recycle time, 2 s ^13^C­{^31^P} REDOR experiments used typical REDOR dephasing times of 6–10 ms, corresponding to 80–100 τ_R_ and MAS rates of 10 or 12.5 kHz, as given in each figure. Broadband TPPM decoupling during signal acquisition was used in all of the ^13^C and 1D ^31^P experiments.

2D ^1^H–^31^P CPMAS heteronuclear correlation experiments were performed with Frequency-Switched Lee–Goldburg (FSLG) decoupling during t1 (^1^H field strength 100 kHz, 0.5 or 1 ms contact time, as given with the figures, 2 s recycle delay). Broadband SPINAL 64 decoupling was used during the t_2_ signal acquisition. Samples were placed in 3.2 mm zirconia rotors and recorded with a Bruker wide-bore, E-free, triple resonance ^1^H–^13^C-^31^P MAS probe.

^13^C spectra were referenced to the glycine Cα signal at 43.1 ppm relative to the TMS signal at 0 ppm. ^31^P spectra were referenced to the hydroxyapatite ^31^P signal at 2.85 ppm relative to 85 wt % H_3_PO_4_ at 0 ppm. ^1^H spectra were referenced to the hydroxyapatite signal at 0 ppm, relative to TMS at 0 ppm.

The HAp-metabolic acid materials were studied using natural abundance ^43^Ca NMR experiments, which were acquired at 20.0 T on a Bruker Avance III-850 (850 MHz ^1^H frequency) spectrometer at the UK 850 MHz Solid-State NMR Facility, with the assistance of Dr Dinu Iuga, operating at a ^43^Ca Larmor frequency of 57.22 MHz, using a low-γ 7 mm Bruker MAS probe spinning at 5 kHz. For the simple OCP phase, a RAPT (rotor assisted population transfer) enhancement scheme was used (offset of 150 kHz, RF ∼9 kHz), followed by a 90° selective solid pulse of 1.5 μs. A total of 137864 transients were acquired, with a recycle delay of 0.5s. For all the intercalated OCP samples, a multi-DFS (double frequency sweep) enhancement scheme followed by a 90° selective pulse of 1.5 μs was used,^67,68^ which was first optimized on a ^43^Ca-labeled CaHPO_4_ sample.^69^ A total of 65500 transients were acquired, with a recycle delay of 0.5 s. A ^43^Ca-enriched HAp phase was also studied at 20.0 T for comparison to the corresponding OCP phases. In this case, a Bruker 4 mm probe was used, and 64 transients were acquired, with a recycle delay of 0.8 s. All ^43^Ca chemical shifts were referenced at 0 ppm to a 1 mol·L^–1^ aqueous solution of CaCl_2_.^70^

### 2D ^1^H–^31^P Spectral Fitting

The intensities of experimental 2D ^1^H–^31^P spectra were normalized so that the maximum intensity equaled 1. Fitting of a linear combination of two or three experimental 2D ^1^H–^31^P correlation NMR spectra of model bone mineral materials to an experimental 2D ^1^H–^31^P correlation NMR spectrum of ex vivo bone was performed in MatLab using the “lsqcurvefit” MatLab function.

Total residuals are calculated as

$$total\;residual=\sqrt{\sum_{i,j}{\left(I_{i,j}^{fit}-I_{i,j}^{bone}\right)}^{2}}$$

where *I*_*i*,__*j*_^*a*^ is the intensity of the *i, j* data point in the 2D spectrum of *a* (= bone or fitted spectrum).

The residual 2D spectrum is calculated as *I*_*i*,__*j*_^fit^–*I*_*i*,__*j*_^bone^ at each *i, j* data point.

### Powder X-ray Diffraction

PXRD experiments were performed on a Philips X’Pert Pro powder diffractometer equipped with an X’celerator RTMS detector using Ni-filtered Cu Kα radiation of wavelength 0.154 nm. Samples were ground into fine powders and mounted on flat steel plates, and data collection was performed over the range 2θ = 3°–60°. Different amounts of the sample were used for the different compounds, and hence, the signal-to-noise ratio of the diffraction patterns differ between samples.

### TEM

TEM imaging of intrafibrillar and extrafibrillar mineral samples was performed on a Talos F200C G2 (Thermo Fisher Scientific, Eindhoven) operating at 200 kV equipped with a Ceta-S camera at the Radboudumc Electron Microscopy Center. The sliced mineralized collagen was transferred on the copper grids (Carbon Square Mesh, 200 mesh, UL, EMS) by using 100% ethanol solution.

TEM imaging of HAp–citrate, HAp–lactate, and HAp–citrate–lactate were performed on a Tecnai G2 80–200 kV transmission electron microscope at the Cambridge Advanced Imaging Centre, operating at 120 or 200 kV.

### SEM

For scanning electron microscopy (SEM) imaging, all samples were coated with carbon to a total thickness of 8 nm by using a Leica ACE600 sputter coater. Imaging was conducted with a Zeiss Crossbeam 550 (Radboudumc Electron Microscopy Center), equipped with a field-emission gun, operating at an acceleration voltage of 3 kV, a probe current of 50 pA, and a constant working distance of 4.5 mm. Secondary electron and in-lens backscattered electron detectors were used to capture all images.

### AFM

A small amount of each of the samples was dispersed into distilled water to allow a separation of individual particles, and then, a drop of the dispersion was placed onto a clean microscope cover glass, which was further allowed to dry on a sealed container with silica for a period of at least 1 week at room temperature in order to reduce to a minimum the hydration conditions of the samples that can modify the results. AFM (Park Systems, XE7) was performed in the spectroscopy mode with an approach piezo speed of 100 nm/s until defined loads were reached, depending on the stiffness of the sample under study. The measurements were made using commercial rectangular AFM cantilevers (*k* = 2.7 N/m, maximum load = 150 nN).

The stiffness of each of the cantilevers used was determined by using Sader’s method,^71^ while the sensitivity was carefully measured after each measurement by using a material with a stiffness several orders of magnitude higher than that of the measured samples. In this case, glass (*E*_glass_ = 70 GPa) was used to adjust the sensitivity by performing approaching and retraction with the AFM cantilever with the same approaching speed (100 nm/s) at the same maximum load levels (20 and 150 nN). With this information, it was possible to obtain the deformation due to the deflection of the cantilever (F/k), which was later subtracted from the information obtained from the measurements on the samples to get the real deformation due to the penetration of the cantilever tip into the sample as

1 h=Z−F/k

The tips of the cantilevers were analyzed using SEM, as shown in Figure 1, finding approximate spherical tips with radius R ≈ 25 nm. The spherical tip of the cantilevers allows the use of the Hertz model to measure the Young’s modulus of the samples using

2 $$F=\frac{3}{4}{{\left(\frac{E}{1-v^{2}}\right)}^{*}\sqrt{R}h}^{3/2}$$

where *E*_sample_ ≪ *E*_tip_, which is valid as the tip is made from silicon, *R* is the tip radius, and *h* is the penetration depth of the tip into the sample. Equation 2 is strictly valid for *h* ≤ 0.4R, leading to a maximum measuring depth for the samples of approximately 10 nm.

Scans on the contact mode at 500 × 500 nm were performed on individual particles, and then, 4 approach/retraction indentations were performed equidistant from each other to measure Young’s modulus with eq 2. Then, calibration of the sensitivity was performed before moving to a different location or particle. The procedure was repeated until at least 50 measurements were obtained for each sample.

Differences in the results between the samples were evaluated by using a multiple range test with a significance of 0.05.

### Solubility Assessment

Three pellets of the same material (HAp–citrate, HAp–lactate, or bone) and same dimensions were formed by pressing powdered samples into a mold. The pellets were suspended at the center of separate 50 mL beakers in glass baskets. Each beaker contained 40 mL of acetic acid buffer (0.087M) at pH 4.0 and tetraphenylphosphonium bromide (∼1.0 mM) as an internal concentration reference. The solution was stirred with a magnetic stirrer at 90 rpm. At each of the following time periods: 5, 15, 25, 50, 90, 180, 300, 600, and 1440 min (unless otherwise stated), one 2.0 mL aliquot of solution was taken for ^31^P solution-state NMR measurements to determine the phosphate ion concentration. A Bruker 500 MHz Avance III HD spectrometer and dual resonance broadband observe probe was used for the ^31^P solution-state NMR measurements at a frequency of 202.48 MHz. The phosphate ion concentration was calculated by integrating the area under the peak and referenced to the concentration of tetraphenylphosphonium bromide, and a total recycle time of 3.3 s was used to ensure full relaxation of the species.

For each pH value, the average of the three phosphate ion concentrations was used to plot the graph in Figure S10 (concentration of phosphate ion against time).

## Supplementary Material



## Notes

Contains SEM and TEM images of collagen fibrils calcified with cHAp (Figure S1); pXRD for all materials (Figure S2); ^31^P of all HAp and OCP materials and starting materials (Figure S3); additional TEM images of the HAp materials (Figure S4); ^43^Ca NMR spectra of the HAp materials (Figure S5); 2D ^1^H–^31^P correlation NMR spectra and analysis for the HAp materials (Figure S6); fits of 2D ^1^H–^31^P NMR spectra of intra- and extrafibrillar models to that of bone for alternative ^1^H–^31^P contact times (Figure S7); ^13^C CPMAS and ^13^C­{^31^P} REDOR NMR spectra for the OCP and HAp materials (Figure S8); AFM data for HAp–citrate and –lactate (Figure S9); and mineral solubility data for HAp–citrate, HAp–lactate, and bone (Figure S10) (PDF)
