# Supplementary material for: Role of Metabolic Acids in Shaping Bone-like Apatite Architectures
Source: Chem Mater. 2026 Mar 26;38(7):3189–201. doi: 10.1021/acs.chemmater.5c02593 (PMC13084994; doi:10.1021/acs.chemmater.5c02593)
Supplement: Supplementary file 1 [file cm5c02593_si_001.pdf]

Supplementary Information

for

Role of metabolic acids in shaping bone-like apatite architectures

Yang Li[1], Rui Li[1], Thomas Kress[1], David G. Reid[1], Karin H. Müller[2], Danielle Laurencin[3], Christian Bonhomme,[4] E. Alex Ossa,[5] Chenglong Li[6], Robin van der Meijden[6], Nico Sommerdijk,[6] and Melinda J Duer[1]*

[1]Department of Chemistry, University of Cambridge, Lensfield Road, Cambridge, CB2 1EW, UK
[2]Cambridge Advanced Imaging Centre, Department of Physiology, Development and Neuroscience, University of Cambridge, Downing Street, Cambridge, CB2 3DY, UK
[3]ICGM, Univ Montpellier, CNRS, ENSCM, Montpellier, France
[4] Laboratoire de Chimie de la Matière Condensée de Paris, UMR Sorbonne Université CNRS, Sorbonne Université, 4, place Jussieu, 75252 Paris Cedex 05, France
[5]Department of Production Engineering, Universidad EAFIT, Cra 49, No 7 sur 50, Medellín, Colombia
[6]Department of Medical BioSciences and Radboud Technology Center – Electron Microscopy Center, Radboud University Medical Center, Geerte Grooteplein, 6525 AG Nijmegen, The Netherlands

Table S1: Elemental and pXRD analyses of the OCP-metabolic acid and HAp-metabolic acid materials in this work. Ca/P ratio and number of carbon per unit cell are calculated from elemental analysis. d_{100} lattice spacings are determined from powder XRD (Fig S1). Carbon, calcium and phosphate compositions are averages from typically three separate syntheses.

Material	Carbon wt%	Approx. no. of carbon atoms per OCP unit cell§	Ca/P ratio*	d_{100} / nm‡
OCP-citrate	3.5	6.2	-	2.15
OCP-lactate	1.7	3.0	-	1.92
HAp-citrate	3.6	-	1.49	2.18
HAp-lactate	2.5	-	1.41	1.87
HAp-citrate-lactate	3.4	-	1.50	-

§ Per unit cell equivalents of the metabolic anion and P content is given for comparison to the original OCP structures, and so a unit cell is assumed to contain 16 Ca^{2+} as in OCP.

* Ca/P ratio for pure OCP is 1.33; for OCP with substitution of one HPO_4^{2-}, the Ca/P ratio is 1.45; with substitution of two HPO_4^{2-}, Ca/P ratio is 1.6. For comparison, the Ca/P ratio for HAp is 1.67 and for the α-tricalcium phosphate (TCP) starting material in the OCP-metabolic acid syntheses, 1.5.

‡ d_{100} for pure OCP is 1.87 nm.

TGA showed typically 10 wt% water in the 10-day synthesis materials, but the water content measured by TGA was highly variable between samples. 10 wt % water corresponds to ~10 molecules of water per unit cell if the initial OCP-metabolic acid double salt chemical composition is assumed.[1,2]

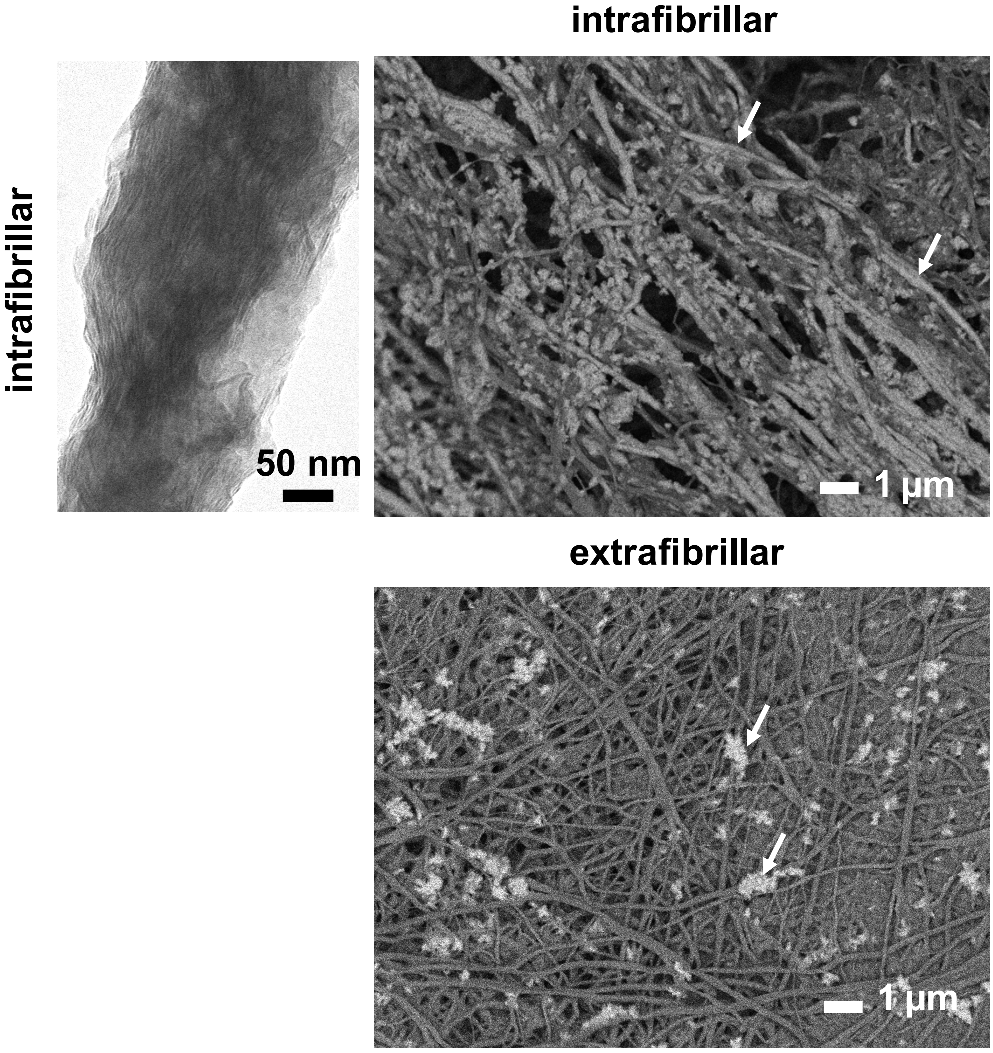

Fig S1: TEM (top left) and BSE SEM (right) images of collagen fibrils calcified with cHAp, cell-free in vitro with and without poly(Asp) generating intra- and extrafibrillar mineral respectively, mimicking in vivo bone mineral morphology but not including the cross-fibrillar mineral observed in mature bone mineral. SEM was used to assess the spatial extent and homogeneity of mineralization across collagen fibrils, while TEM was used to examine mineral morphology at the nanoscale.

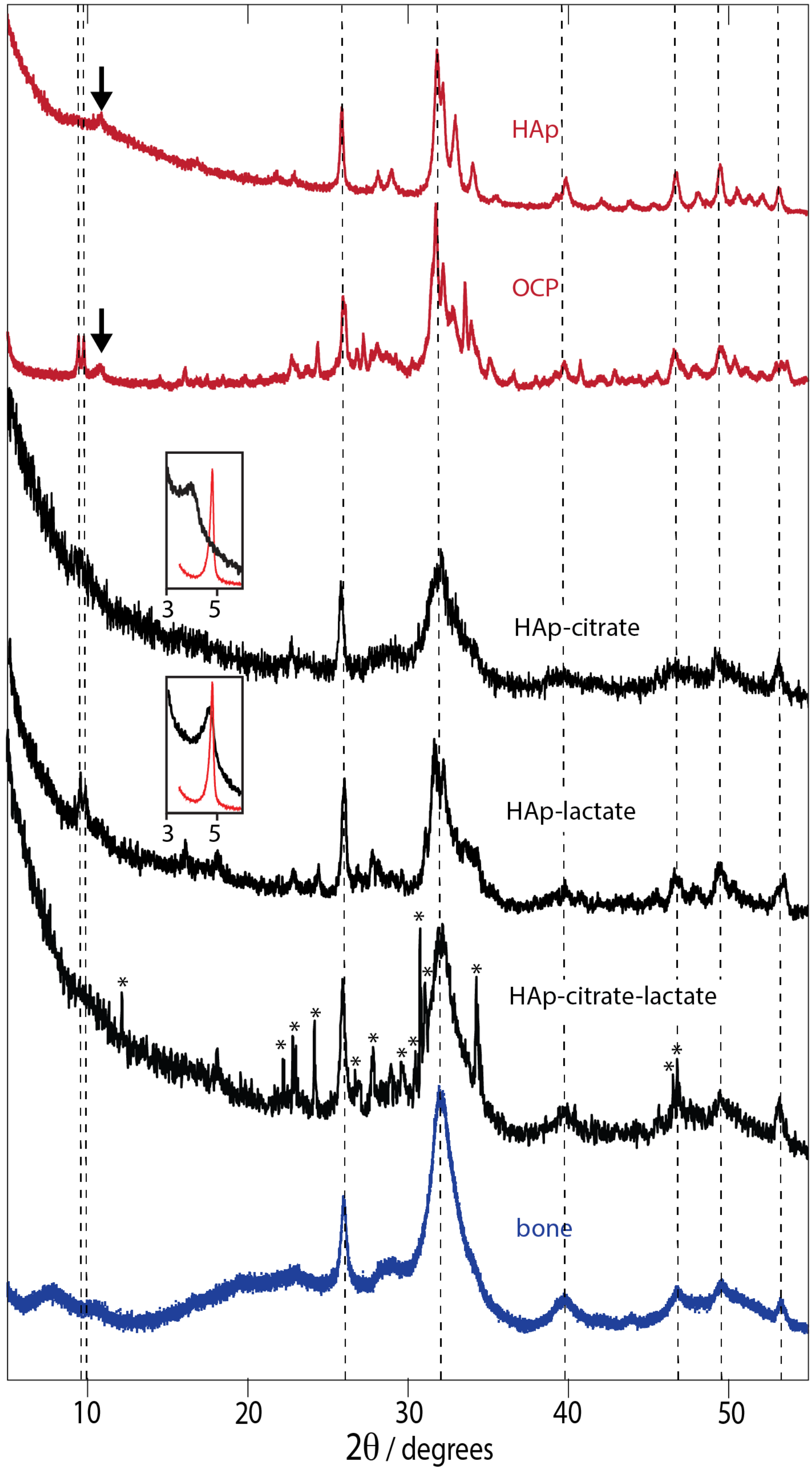

Fig S2: pXRD diffraction patterns for the OCP-metabolic acid double salts (2-day syntheses) and the HAp-metabolic acid materials. Insets: the (low angle) (100) reflection (black) compared to that for pure OCP (red); horizontal scales are 2θ in degrees. Dotted lines indicate the strongest reflections from bone (mineral) and two reflections around 2θ ~10° that are distinctive

for the OCP crystal structure ((1-10) and (010)). Arrows in the patterns for HAp and OCP indicate the putative HAp peak in the OCP diffraction pattern. The diffraction pattern for HAp-citrate-lactate material has residual (sharp) reflections from α-TCP starting material; the expected positions of these are marked with *. The insets show expansions of the d_{100} reflection position. The pXRD patterns of both OCP-citrate and OCP-lactate double salts exhibit the characteristic OCP low angle (100) reflection between $2\theta \sim 4 - 5°$ ($2\theta = 4.839°$ for OCP; see Figure 2 insets) and strong OCP-like (1-10) and (010) reflections around $2\theta \sim 10°$ ($2\theta = 9.599°$, $9.831°$ for OCP).

The d_{100} spacings are larger in OCP-citrate than pure OCP (OCP d_{100} = 1.87 nm; OCP-lactate d_{100} = 1.92 nm; OCP-citrate d_{100} = 2.15 nm; see Table S1) consistent with incorporation of the associated metabolic acid anion into the OCP hydrated layer. The d_{100} spacing for OCP-citrate is larger than that for OCP-lactate consistent with citrate being a larger anion than lactate. Samples of pure OCP typically contain some small amount of HAp.[3,4] That is the case here too, evidenced, by a small, broad HAp (100) reflection in the pXRD pattern for the OCP sample at $2\theta \sim 10.9°$ (other expected HAp reflections overlap with the relatively broad OCP reflections).

The pXRD pattern for the HAp-lactate, the pXRD pattern still contains an OCP-like (100) (see inset in figure), (1-10) and (010) reflections. The d_{100} spacing for the HAp-lactate material is smaller than in the initial OCP-lactate double salt and the pXRD reflections for this material are generally broadened compared to the reflections for the OCP-lactate double salt, particularly in the $2\theta \sim 30° - 35°$ range where some of the more distinctive reflections that would distinguish an HAp or OCP-like structure are expected. Above $2\theta \sim 30°$, the differences in the pXRD patterns for pure HAp and OCP are subtle. This along with the broadening of reflections means that between $2\theta \sim 30° - 60°$, the HAp-lactate material pXRD pattern resembles both the HAp and OCP pXRD patterns; only the distinctive OCP-like (100), (1-10) and (010) reflections at low 2θ suggest predominant retention of OCP structural characteristics.

For the HAp-citrate material, a distinctive, though broad, OCP-like (100) reflection remains at low angle. The associated average d_{100} spacing is slightly larger than for the OCP-citrate double salt (2.18 nm compared to 2.15 nm in the OCP-citrate double salt, Table S1). There are no other reflections that can be confidently assigned to specifically OCP-like structures or HAp. The reflections are broad so that as in the case for the HAp-lactate material, the pXRD pattern for HAp-citrate resembles those for both HAp and OCP above $2\theta \sim 30°$.

The HAp-citrate-lactate-containing synthesis product did not show any low-angle reflections consistent with OCP-like (100), (1-10) and (010) reflections and the pXRD pattern for this material overall resembles more that for disordered/ nanocrystalline HAp than an OCP-like material. Some weak reflections from the α-TCP starting material are often present in this diffraction pattern, indicating that there has been incomplete reaction of the α-TCP starting material.

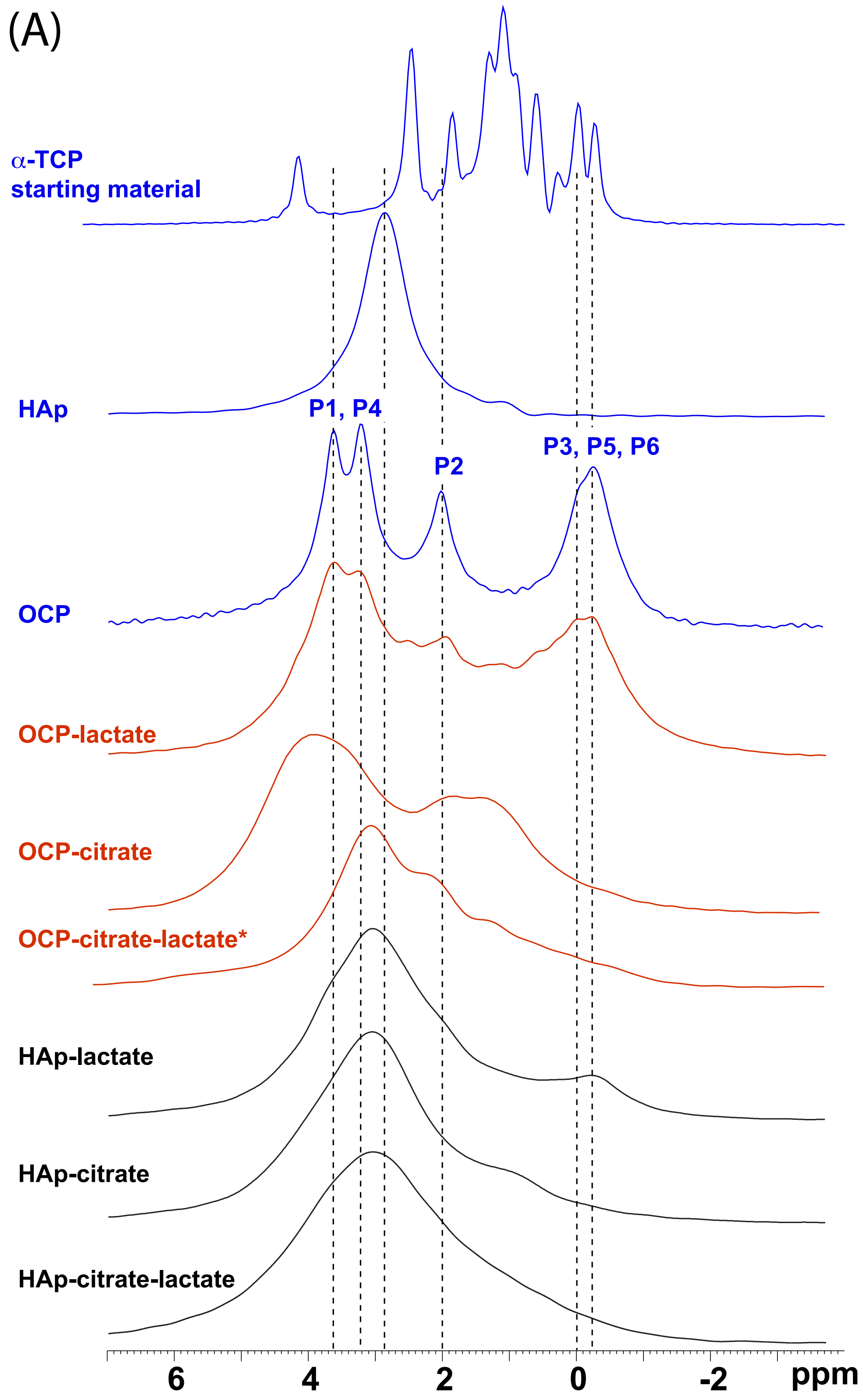
(A)
α-TCP
starting material
HAp
P1, P4
P2
P3, P5, P6
OCP
OCP-lactate
OCP-citrate
OCP-citrate-lactate*
HAp-lactate
HAp-citrate
HAp-citrate-lactate
6
4
2
0
-2
ppm

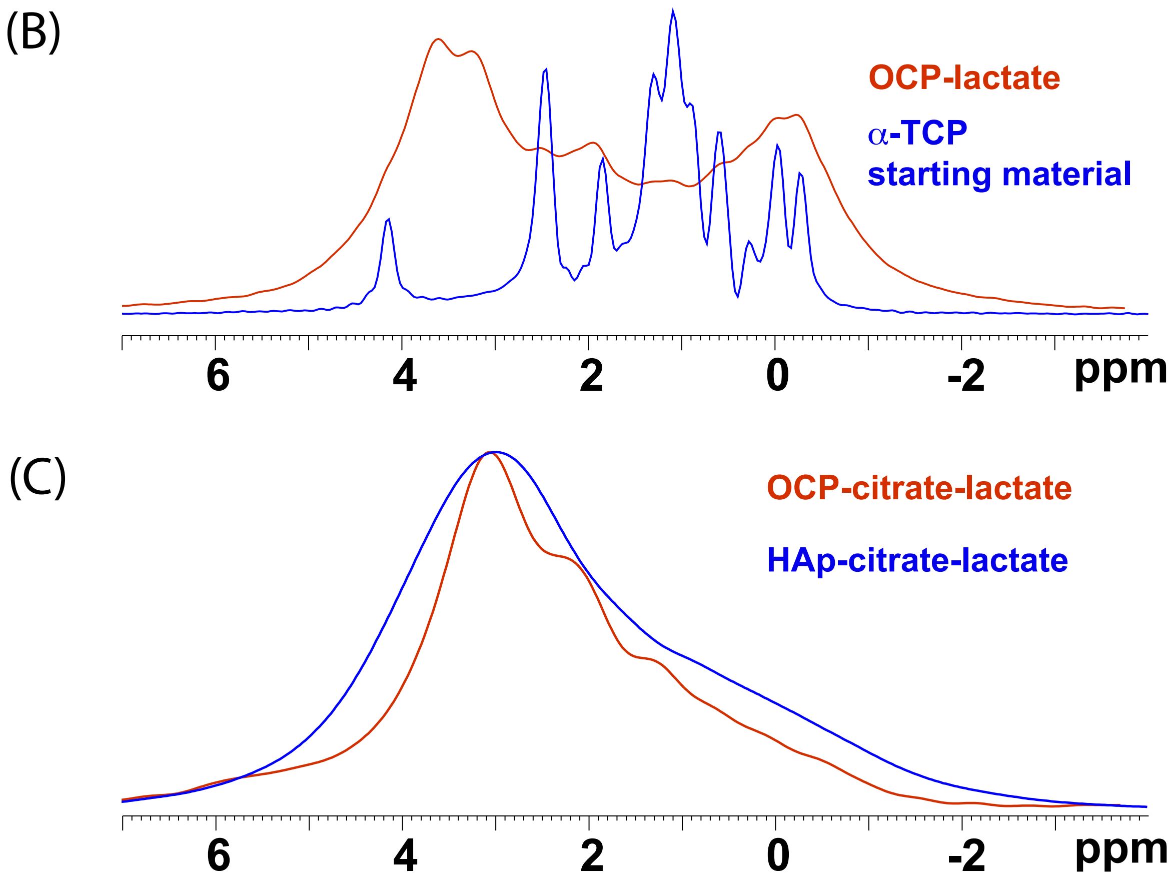

Figure S3: (A) Comparison of the ^{31}P direct polarization (DP) magic-angle spinning (MAS) NMR spectra of the OCP-metabolic acid double salts and the HAp-metabolic acid materials investigated in this work. * ^{31}P cross-polarization (CP) MAS NMR spectrum; only small amounts of OCP-citrate-lactate could be produced with the α-TCP starting material giving the dominant signals by DP ^{31}P NMR, hence the ^{31}P CP NMR spectrum is shown here, because signals from α-TCP are not observed by cross polarization. Sample spinning rate is 10 kHz for all samples. ^{31}P DP spectra for pure HAp (nanocrystalline) and OCP are shown for reference at the top. Dotted lines indicate the chemical shifts of the characteristic HAp and OCP ^{31}P signals. Note that the spectrum for the OCP-lactate double salt also shows residual signals from the α-TCP starting material used in the synthesis (see Fig S2B for overlay of the OCP-lactate and α-TCP ^{31}P spectra). OCP assignments refer to the phosphate labelling in Fig 1. **(B)** Overlay of the ^{31}P direct polarization spectra for the OCP-lactate sample and the α-TCP starting material, showing that there are residual signals from the starting material present in the OCP-lactate sample. **(C)** Overlay of the ^{31}P{^{1}H} CP spectra for the OCP-citrate-lactate and HAp-citrate-lactate materials for comparison.

The ^{31}P DP MAS spectrum for the OCP-citrate double salt has been reported and assigned previously:[1,5] in summary, the highest frequency ^{31}P chemical shifts (signal/s in the range 3.1 – 3.6 ppm) are from the OCP apatitic orthophosphate groups (P1, P4, see Fig 1 for phosphate labelling), the lowest frequency signals (broad, poorly resolved set of signals, 0 - –1 ppm) are from the hydrated layer HPO_4^{2-} / PO_4^{3-} (P3 (PO_4^{3-}); P5, P6 (HPO_4^{2-})) and the intermediate frequency signal (1.8 ppm for OCP-citrate, 2.0 ppm in pure OCP) is from the orthophosphate groups in the interface between the OCP apatitic-like and hydrated layers (P2). The OCP-lactate double salt gives a similar distribution of ^{31}P chemical shifts, and we assign them similarly. In the CP ^{31}P spectrum for OCP-citrate-lactate, the peak maximum in the

orthophosphate region (3.05 ppm) is between that expected for OCP-like (3.1 – 3.6 ppm) and HAp (2.85 ppm) orthophosphate sites, and is similar to that expected for nanocrystalline HAp.[6–10] Broad signals centred at ~2.2 and ~1.3 ppm, plus the low frequency “tail” in the spectrum cover the spectral region for OCP-like hydrated layer HPO_4^{2-} and PO_4^{3-} and hydrated layer-apatitic layer interface region, suggesting there are OCP-like structures in the material. The CP relative intensities are not quantitative so we cannot deduce the relative amounts of the different OCP/ HAp phosphatic environments from this spectral analysis.

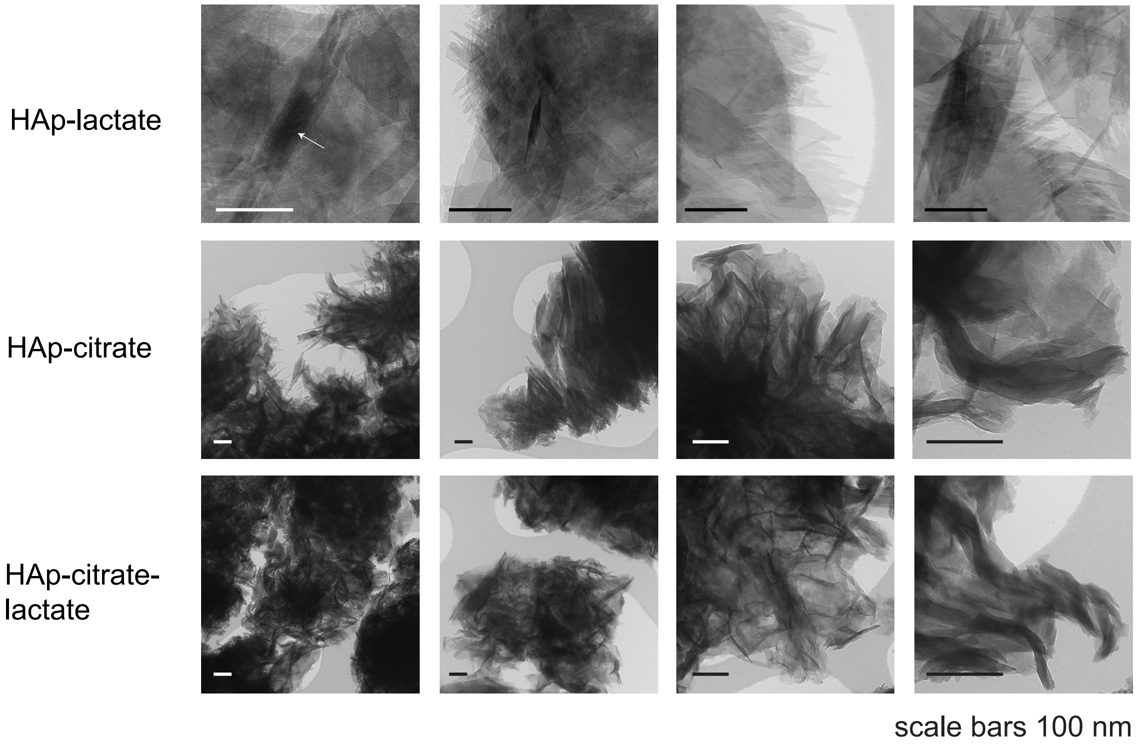

Figure S4: Additional TEM images of HAp-metabolic acid materials to characterize mineral platelet morphology and interlayering. For the HAp-lactate material the white arrow indicates a particle that exhibits interlayering. All scale bars, 100 nm. To note, any remaining α-TCP crystals in the mixed citrate-lactate samples are expected to be much larger than the length scales represented by the sizes of the TEM images; the pXRD reflections from α-TCP crystals/ domains in these samples are very sharp (Fig S1) corresponding to crystals with sizes much larger than the few hundred nanometer image dimensions here.

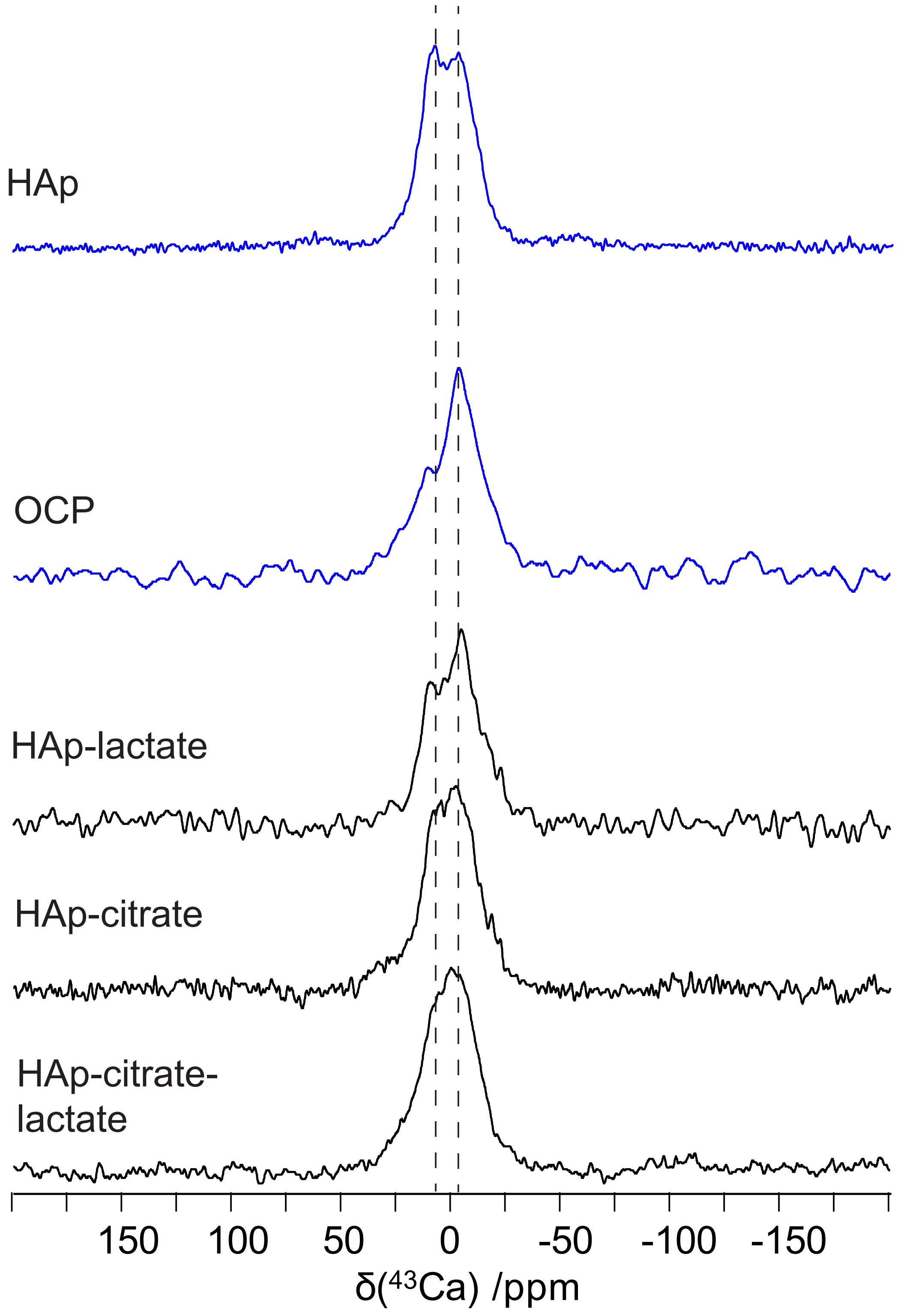

Figure S5: ^{43}Ca double-frequency sweep (DFS) MAS NMR of the HAp-metabolic acid materials. Spectra for pure OCP and HAp are shown for comparison. Dotted lines are for guidance only, and indicate the main features of the HAp ^{43}Ca spectrum at this magnetic field.

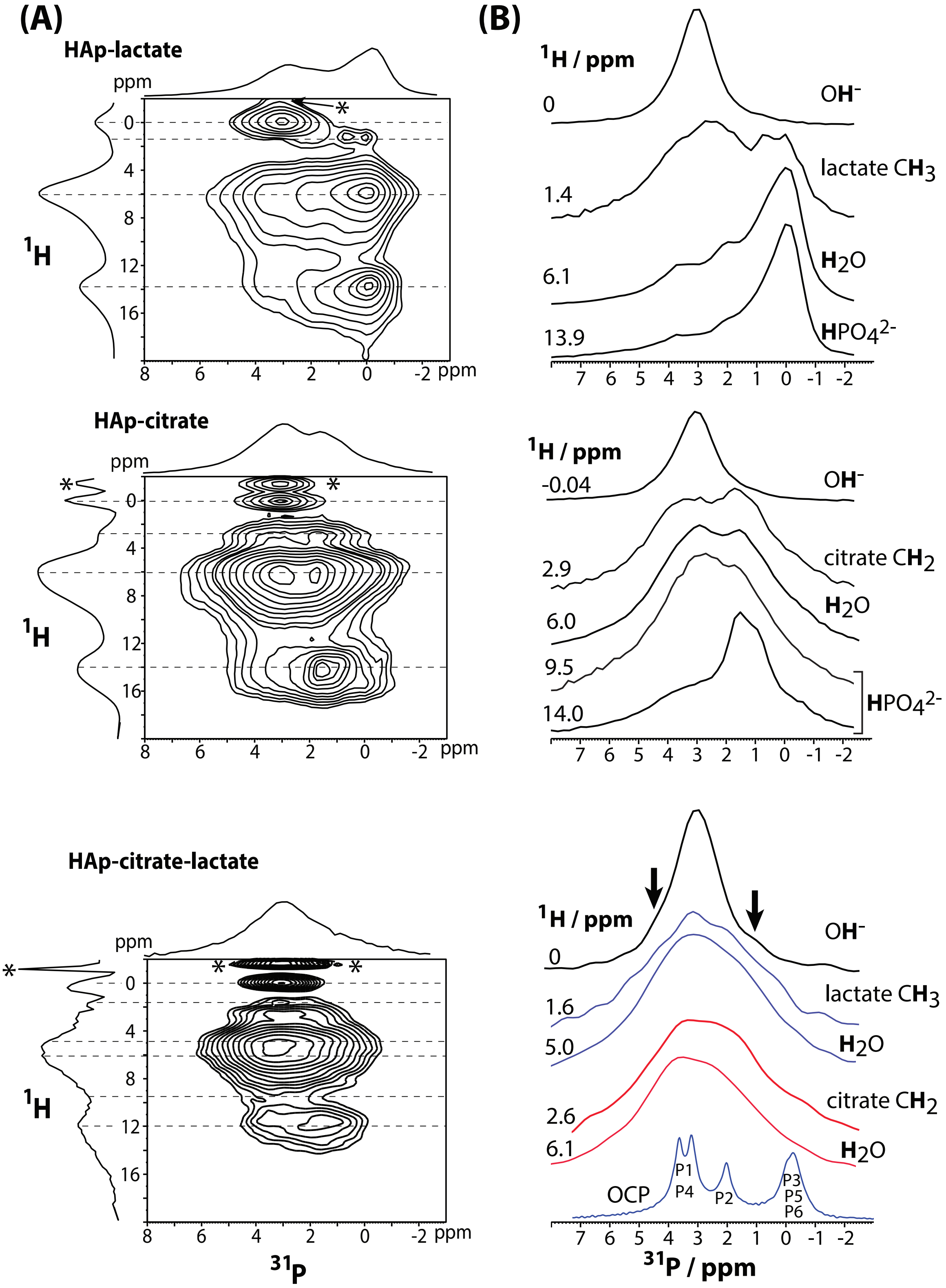

Fig S6: (A) 2D ^{1}H-^{31}P correlation spectra for the HAp-metabolic acid materials synthesized in this work, ^{1}H-^{31}P contact time 1 ms. **(B)** ^{31}P spectra from slices through the corresponding 2D ^{1}H-^{31}P correlation spectrum at the ^{1}H chemical shift indicated.

The 2D ^{1}H-^{31}P correlation spectrum for the HAp-lactate material shows a ^{1}H 0 ppm – ^{31}P 3.1 ppm correlation consistent with HAp structures, ^{1}H 0 ppm corresponding to the HAp OH^- ^{1}H signal and ^{31}P 3.1 ppm to HAp orthophosphate in nanocrystalline material. [6–10] The broadness

of the HAp ^{31}P signal compared to that typical of crystalline HAp (e.g. see Fig S2), indicates that the HAp orthophosphate sites are disordered or heterogeneous, as would be expected for nanoscopic-sized domains. There is also an unequivocal ^{1}H signal from HPO_4^{2-} centred at 13.9 ppm. This ^{1}H signal is correlated with ^{31}P signals (~3.5, ~2, ~0 ppm), that may be assigned to remaining OCP-lactate-like environments (see the OCP-lactate only synthesis spectrum in Fig S2). The water ^{1}H signal at 6.1 ppm is consistent with water in an OCP-like hydrated layer or other confined water layers; by way of comparison, water molecules on external surfaces of nanocrystalline HAp have a ^{1}H chemical shift of 4.85 ppm.[11] The 6.1 ppm water ^{1}H signal correlates with a similar intensity distribution of ^{31}P signals as the HPO_4^{2-} ^{1}H signal, showing that the water is in a similar environment to the HPO_4^{2-}, namely an OCP-like hydrated layer. The lactate methyl ^{1}H signal at 1.4 ppm shows correlation with a very broad/ poorly resolved set of ^{31}P signals covering the expected range for HAp- and OCP-like structures. Importantly, there is a much stronger correlation with the ^{31}P chemical shift range for apatitic orthophosphate (2.8 – 3.6 ppm) than would be expected if the lactate were confined to an OCP-like hydrated layer; if it were, we would expect an intensity distribution in the spectral slice more similar to those for the HPO_4^{2-} and water ^{1}H spectral slices. We suggest that at least some lactate is at interfaces with HAp, resulting in relatively strong spectral correlation between lactate ^{1}H and HAp-like orthophosphate ^{31}P. Thus, we conclude that the HAp-lactate material is dominated by molecular structures similar to the OCP-lactate double salt structure with some additional HAp-like regions, consistent with the pXRD, TEM with lactate probably present near HAp domains.

The 2D ^{1}H-^{31}P correlation spectrum for the HAp-citrate material shows a similar ^{1}H (0 ppm) – ^{31}P (3.1 ppm) correlation signal, consistent with nanocrystalline hydroxyapatite structures. The ^{31}P lineshape in this correlation is broad as for the HAp-lactate material, consistent with nanoscopic-sized HAp domains which TEM confirms exist in this material (Fig S3). The broad ^{1}H signal centred at 14.2 ppm is due to ^{1}H in HPO_4^{2-} anions, confirmed by the most intense ^{31}P signals correlated with them being between 0.9 – 1.5 ppm, the expected chemical shift range for HPO_4^{2-}. There are also the expected weaker correlations to ^{31}P signals above 3 ppm consistent with apatitic-like PO_4^{3-} that are more distant from the HPO_4^{2-} ^{1}H, and therefore exhibit weaker spectral correlations to hydrated layer ^{1}H. There is ^{1}H signal intensity in the range 9 – 10 ppm consistent with HPO_4^{2-} ^{1}H on the surface of nanocrystalline HAp.[8,12] ^{1}H signals in this chemical shift range correlate with a broad range of ^{31}P chemical shifts and this is discussed in more detail below in the context of the HAp-citrate-lactate material where the equivalent signal is more intense. The citrate CH_2 ^{1}H signal at 2.9 ppm[1] is correlated with a set of broad overlapping ^{31}P signals covering the chemical shift range for OCP-citrate and HAp structures, consistent with citrate being both in OCP-citrate-like hydrated layers and at internal hydrated layer interfaces with HAp as for the lactate material. The ^{1}H signal for water is centred at 6.0 ppm, but broad and asymmetric, suggesting multiple water environments. A ^{1}H chemical shift of 6 ppm is consistent with the water ^{1}H signal in confined layers, and this water ^{1}H signal correlates strongly with the ^{31}P chemical shift range for apatitic orthophosphate (2.8 – 3.6 ppm) suggesting that a considerable proportion of this water signal comes from the hydrated interlayers between HAp layers in the HAp-citrate material observed in TEM (Fig S3).

The 2D ^{1}H-^{31}P correlation spectrum for the HAp-citrate-lactate material again has a characteristic ^{1}H 0 ppm – ^{31}P 3.1 ppm correlation signal consistent with a nanocrystalline HAp component. Importantly, there is also an observable correlation between the HAp OH^- ^{1}H signal and a broad ^{31}P shoulder at ~ 1.2 ppm, a ^{31}P chemical shift similar to those for the hydrated orthophosphate and HPO_4^{2-} in hydrated layers in the HAp-citrate material (B, 0 ppm ^{1}H slice, arrow) consistent with HAp domains interfacing with OCP-like hydrated layers as for the citrate-only material. There is ^{1}H signal characteristic of HPO_4^{2-} from ~9 – 14 ppm, suggesting multiple HPO_4^{2-} populations. The lower frequency HPO_4^{2-} ^{1}H signal range (9 – 11 ppm; Fig S3B) consistent with HPO_4^{2-} on HAp surfaces,[8,12] correlates with a broad ^{31}P signal centred at ~ 3 ppm, characteristic of (disordered) HAp-like orthophosphate and with significant spectral intensity to higher chemical shift including a putative broad shoulder at ~5.5 ppm (B, 9.5 ppm ^{1}H

slice, arrow) which is the expected ^{31}P chemical shift range for HPO_4^{2-} on HAp surfaces.[8,12] The higher ^1H chemical shifts in the HPO_4^{2-} signal region (12 - 14 ppm) are consistent with HPO_4^{2-} ^1H and correlate with a set of broad poorly resolved ^{31}P signals centred at: ~3.7 ppm, ~2.7 ppm and ~1.4 ppm. The 3.7 and 2.7 ppm signals are consistent with apatitic-like orthophosphate and/ or HPO_4^{2-} substitutions in HAp (expected to be ~2.5 – 5.5 ppm).[8,12]

The water ^1H signal for the HAp-citrate-lactate material has multiple components. The lower water ^1H chemical shifts are similar to the (external) surface water ^1H chemical shifts for nanocrystalline HAp (~4.85 ppm)[7] and correlate with ^{31}P signals centred ~2.9 ppm suggestive of HAp-like orthophosphate, e.g. ^1H 4.8 ppm in (B) for this material; thus we assign the lower range of water chemical shifts (below ~5 ppm) to external surface water on HAp domains. Higher water ^1H chemical shifts, e.g. 6.1 ppm in (B) for this material, correlate with a broad range of signals between ~0 ppm to over 5 ppm, but most strongly ^{31}P chemical shifts between ~2.5 and ~3.7 ppm; we assign these higher water ^1H chemical shifts to water in the confined layers between HAp layers in this interlayered material.

The citrate CH_2 and lactate methyl ^1H signals (~2.6 and ~1.6 ppm respectively) overlap with the expected ^1H chemical shifts for HAp structured surface water (H_2O substituting for OH^- and HAp surfaces) and HPO_4^{2-} substitutions in HAp, as well as some overlap with the broad water ^1H signals for this material. That there is significant ^1H signal intensity at the ^1H chemical shifts expected for citrate and lactate ^1H demonstrates that both are present in the mineral structure.

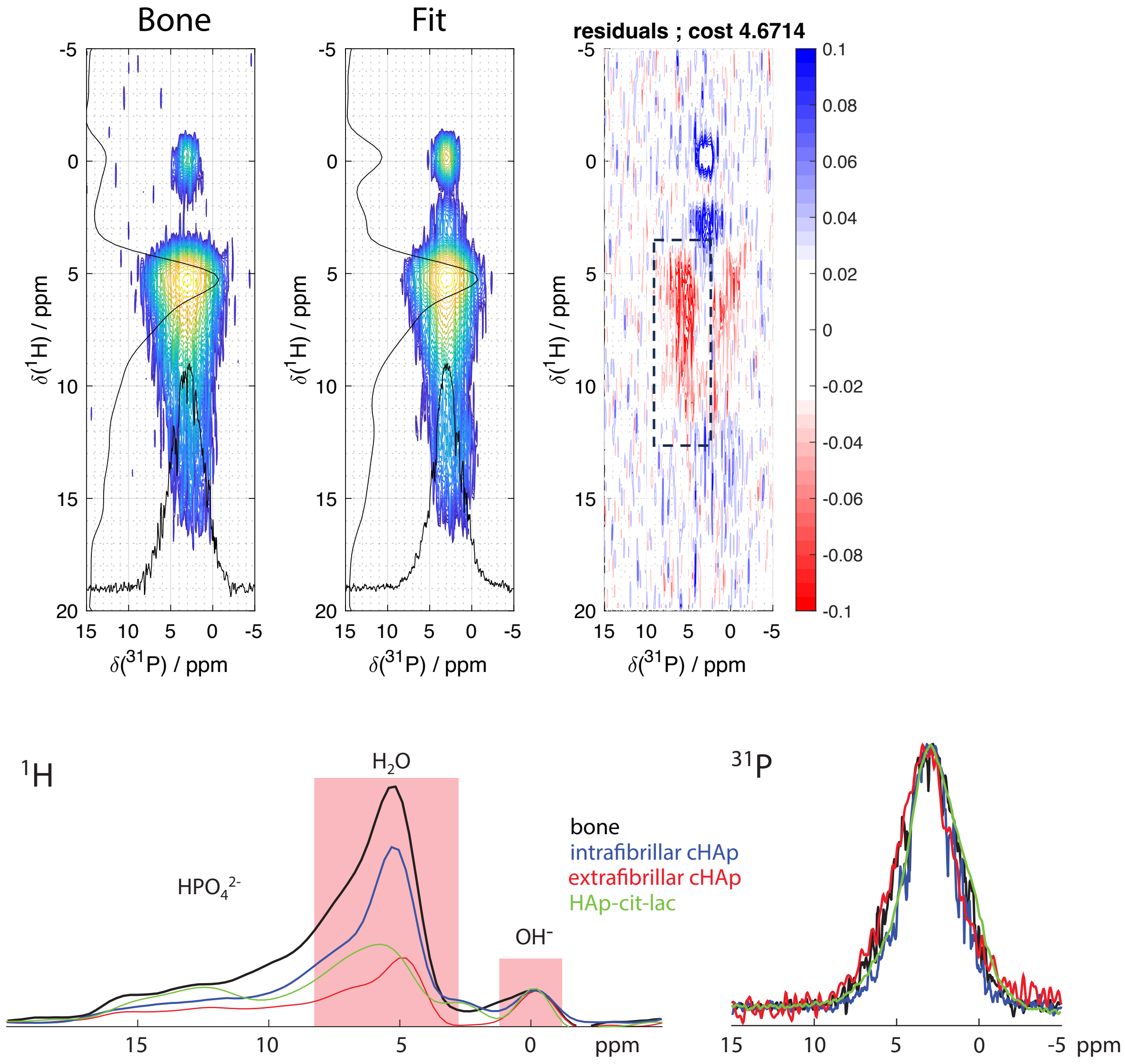

Fig S7: Top: Best fit of 2D ^{1}H-^{31}P correlation NMR spectrum (500 μs mixing time) of air-dried, ground mouse limb bone by a linear combination of the spectra from intrafibrillar cHAp, extrafibrillar cHAp (see Fig 1C) and HAp-citrate-lactate (see Fig 2C). The difference between the bone and fit spectra (right) shows that there is signal intensity missing from a specific type of phosphatic site (dotted rectangle). Bottom: ^{1}H and ^{31}P projections from the 2D ^{1}H-^{31}P spectra of bone and the synthetic models. The intensity of the ^{1}H spectra are normalized to that of the HAp OH^- ^{1}H signal (-0.03 ppm). The intensity of the ^{31}P spectra are normalized so that all spectra have the same maximum intensity.

Neither the bone ^{1}H nor ^{31}P signal intensity can be reproduced by any of the synthetic models alone. The cHAp models lack ^{1}H intensity throughout the water and HPO_4^{2-} ^{1}H region compared to the bone spectrum. The HAp-citrate-lactate model H_2O ^{1}H signal is dominated by higher chemical shift components (>~6 ppm), corresponding to strongly bound water. This model also has significant ^{1}H intensity around ~12 ppm from HPO_4^{2-}, present in the bone spectrum, but under-represented in spectra from the cHAp models.

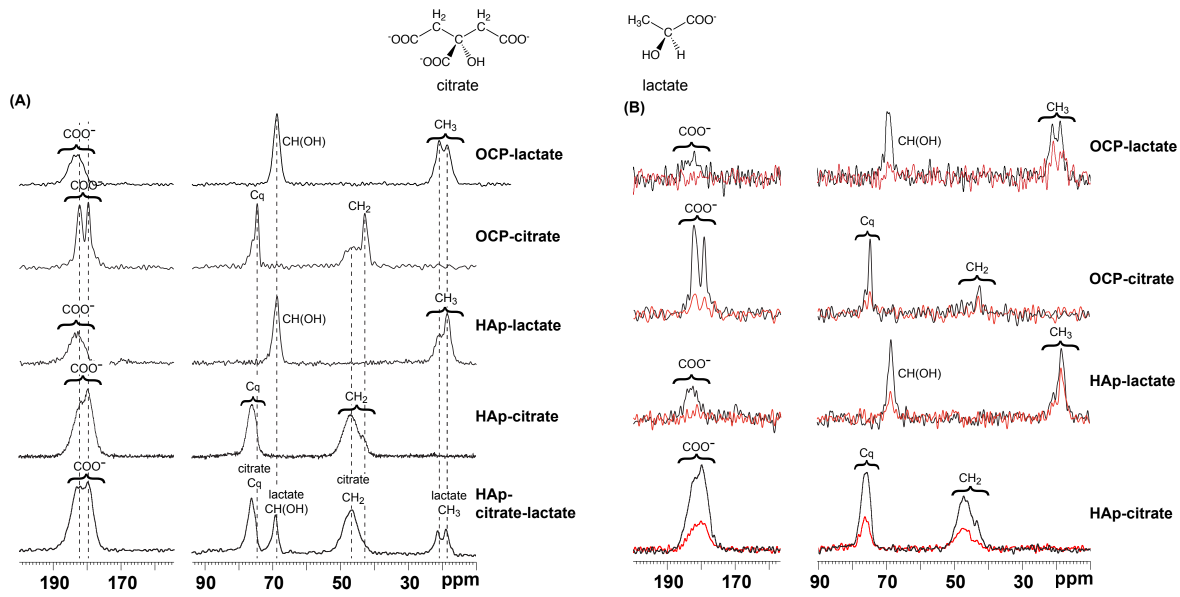

Figure S8: (A) ^{13}C CP MAS spectra of the OCP-metabolic acid double salts and HAp-metabolic acid materials characterised in this work. Signals from citrate and lactate (structures at top) are assigned in the respective spectra. Dotted lines indicate the chemical shifts of representative ^{13}C signals for the OCP-metabolic acid double salts; there are subtle chemical shift differences between these and the HAp-metabolic acid materials. **(B)** $^{13}C\{^{31}P\}$ REDOR spectra of the OCP-metabolic acid double salts and HAp-metabolic acid materials. The spinning rate for all samples was 10 kHz, except the OCP-citrate sample for which it was 12.5 kHz. REDOR dephasing times are 98 τ_R for the OCP-citrate double salt, 100 τ_R for all other samples. Note that the low signal to noise ratio in the $^{13}C\{^{31}P\}$ REDOR spectrum of OCP-citrate here is due to the short ^{13}C T_2 in this material. In this figure C_q refers to the quaternary carbon of the citrate ion.

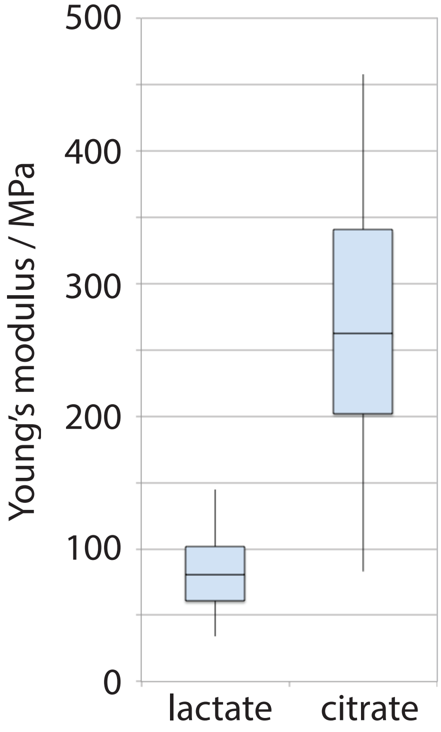

Figure S9: AFM-derived stiffness modulus for the HAp-citrate and HAp-lactate materials.

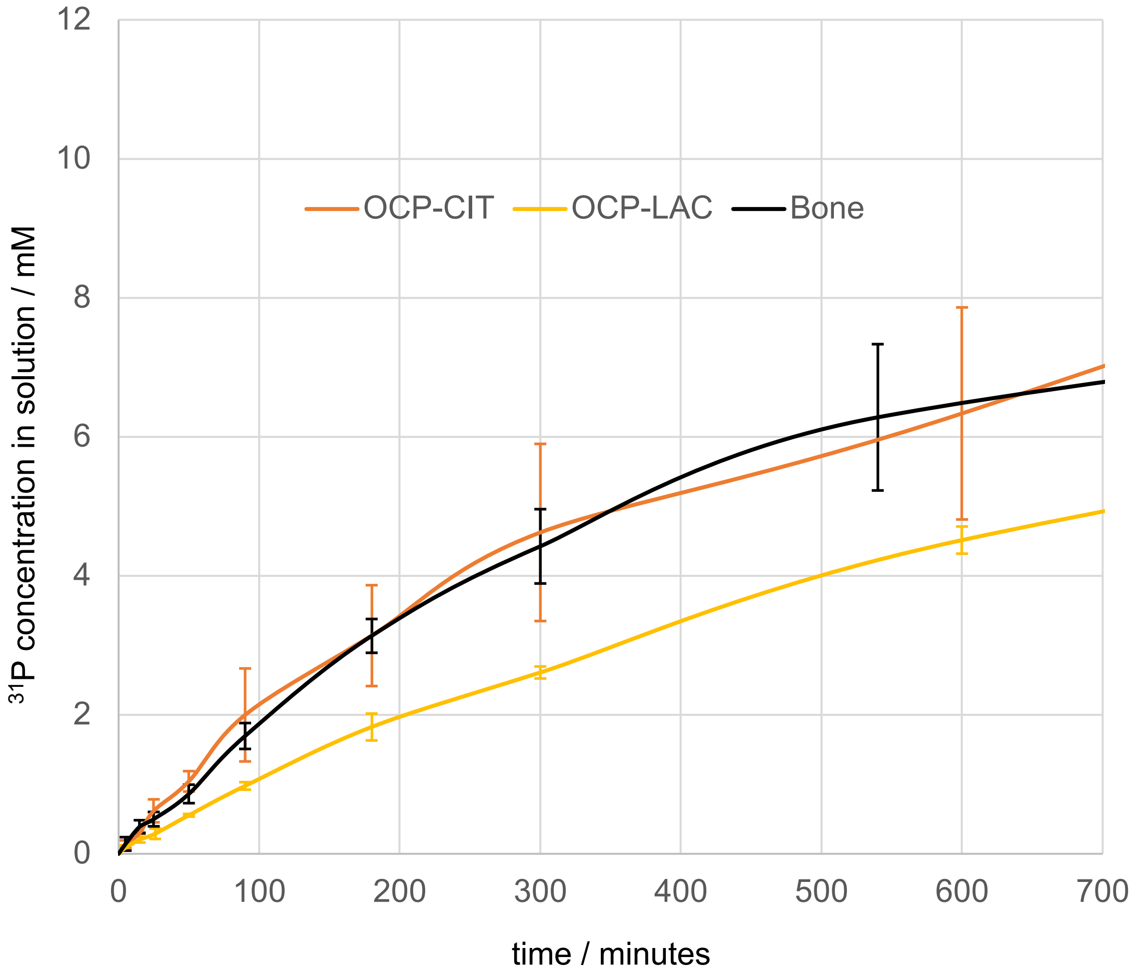

Figure S10. Solubility of HAp-citrate, HAp-lactate and bone mineral at pH 4. Ground powders of each material were pressed into pellets of equal dimensions and immersed in acetic acid at pH 4.0. Orthophosphate concentrations were measured at time points from the intensity of the ^{31}P NMR signal in solution state-NMR spectra.

Supplementary references

(1) Davies, E.; Müller, K. H.; Wong, W. C.; Pickard, C. J.; Reid, D. G.; Skepper, J. N.; Duer, M. J. Citrate Bridges between Mineral Platelets in Bone. *Proc. Natl. Acad. Sci. U. S. A.* **2014**, *111* (14).

(2) Markovic, M.; Fowler, B. O.; Brown, W. E. Octacalcium Phosphate Carboxylates. 2. Characterization and Structural Considerations. *Chem. Mater.* **1993**, *5* (10), 1406–1416.

(3) Tseng, Y.-H.; Zhan, J.; Lin, K. S. K.; Mou, C.-Y.; Chan, J. C. C. High Resolution 31P NMR Study of Octacalcium Phosphate. *Solid State Nucl. Magn. Reson.* **2004**, *26* (2), 99–104.

(4) Davies, E.; Duer, M. J.; Ashbrook, S. E.; Griffin, J. M. Applications of NMR Crystallography to Problems in Biomineralization: Refinement of the Crystal Structure and 31P Solid-State NMR Spectral Assignment of Octacalcium Phosphate. *J. Am. Chem. Soc.* **2012**, *134* (30), 12508–12515.

(5) Nelson, A.; Papawassiliou, W.; Paul, S.; Hediger, S.; Hung, I.; Gan, Z.; Venkatesh, A.; Franks, W. T. T.; Smith, M. E.; Gajan, D.; et al. Temperature-Induced Mobility in Octacalcium Phosphate Impacts Crystal Symmetry: Water Dynamics Studied by NMR Crystallography. *Faraday Discuss.* **2024**, *255*, 451–482.

(6) Gunawidjaja, P. N.; Izquierdo-Barba, I. Solid-State 31P and 1H NMR Investigations of Amorphous and Crystalline Calcium Phosphates Grown Biomimetically From a Mesoporous Bioactive Glass. *J. Phys. Chem. C* **2011**, *115*, 20572–20582.

(7) Wang, Y.; Von Euw, S.; Fernandes, F. M.; Cassaignon, S.; Selmane, M.; Laurent, G.; Pehau-Arnaudet, G.; Coelho, C.; Bonhomme-Coury, L.; Giraud-Guille, M.-M.; et al. Water-Mediated Structuring of Bone Apatite. *Nat. Mater.* **2013**, *12* (12), 1144–1153.

(8) Osman, M. Ben; Diallo-garcia, S.; Herledan, V.; Brouri, D.; Yoshioka, T.; Kubo, J.; Millot, Y. Discrimination of Surface and Bulk Structure of Crystalline Hydroxyapatite Nanoparticles by NMR. *J. Phys. Chem. C* **2015**, *119*, 23008–23020.

(9) Euw, S. Von; Ajili, W.; Delices, A.; Laurent, G.; Babonneau, F.; Nassif, N.; Azaïs, T. Acta Biomaterialia Amorphous Surface Layer versus Transient Amorphous Precursor Phase in Bone – A Case Study Investigated by Solid-State NMR Spectroscopy. *Acta Biomater.* **2017**, *59*, 351–360.

(10) Euw, S. Von; Paquis, C.; Haye, B.; Pehau-arnaudet, G.; Babonneau, F.; Azaïs, T.; Nassif, N. Organization of Bone Mineral : The Role of Mineral – Water Interactions. *Geosciences* **2018**, *8*, 466–484.

(11) Wang, Y.; Azaïs, T.; Robin, M.; Vallée, A.; Catania, C.; Legriel, P.; Pehau-Arnaudet, G.; Babonneau, F.; Giraud-Guille, M. M.; Nassif, N. The Predominant Role of Collagen in the Nucleation, Growth, Structure and Orientation of Bone Apatite. *Nat. Mater.* **2012**, *11* (8), 724–733.

(12) Edén, M. Structure and Formation of Amorphous Calcium Phosphate and Its Role as Surface Layer of Nanocrystalline Apatite: Implications for Bone Mineralization. *Materialia* **2021**, *17* (April), 101107.
